# ATRX limits the accessibility of histone H3-occupied HSV genomes during lytic infection

**DOI:** 10.1371/journal.ppat.1009567

**Published:** 2021-04-28

**Authors:** Joseph M. Cabral, Camille H. Cushman, Catherine N. Sodroski, David M. Knipe

**Affiliations:** 1 Department of Microbiology, Blavatnik Institute, Harvard Medical School, Boston, Massachusetts, United States of America; 2 Program in Virology, Harvard Medical School, Boston, Massachusetts, United States of America; 3 Department of Medical Oncology, Dana-Farber Cancer Institute, Boston, Massachusetts, United States of America; Wistar Institute, UNITED STATES

## Abstract

Histones are rapidly loaded on the HSV genome upon entry into the nucleus of human fibroblasts, but the effects of histone loading on viral replication have not been fully defined. We showed recently that ATRX is dispensable for *de novo* deposition of H3 to HSV genomes after nuclear entry but restricted infection through maintenance of viral heterochromatin. To further investigate the roles that ATRX and other histone H3 chaperones play in restriction of HSV, we infected human fibroblasts that were systematically depleted of nuclear H3 chaperones. We found that the ATRX/DAXX complex is unique among nuclear H3 chaperones in its capacity to restrict ICP0-null HSV infection. Only depletion of ATRX significantly alleviated restriction of viral replication. Interestingly, no individual nuclear H3 chaperone was required for deposition of H3 onto input viral genomes, suggesting that during lytic infection, H3 deposition may occur through multiple pathways. ChIP-seq for total histone H3 in control and ATRX-KO cells infected with ICP0-null HSV showed that HSV DNA is loaded with high levels of histones across the entire viral genome. Despite high levels of H3, ATAC-seq analysis revealed that HSV DNA is highly accessible, especially in regions of high GC content, and is not organized largely into ordered nucleosomes during lytic infection. ATRX reduced accessibility of viral DNA to the activity of a TN5 transposase and enhanced accumulation of viral DNA fragment sizes associated with nucleosome-like structures. Together, these findings support a model in which ATRX restricts viral infection by altering the structure of histone H3-loaded viral chromatin that reduces viral DNA accessibility for transcription. High GC rich regions of the HSV genome, especially the S component inverted repeats of the HSV-1 genome, show increased accessibility, which may lead to increased ability to transcribe the IE genes encoded in these regions during initiation of infection.

## Author summary

The genome of herpes simplex virus (HSV) is subject to host cell processes that organize DNA into ordered structures, known as chromatin, and function to regulate gene expression. Shortly after entry to the host cell nucleus, HSV DNA becomes associated with histones, protein subunits of nucleosomes around which DNA is wrapped. We evaluated the ability of known histone regulatory proteins to load histones onto HSV DNA. Only combinatorial depletion of these factors could reduce the levels of histones on viral DNA, revealing that multiple histone chaperone pathways promote chromatin loading on HSV DNA. However, one of the histone-loaders, ATRX, was significantly more potent in restricting HSV replication than the other factors. While ATRX is widely known for its role in maintaining cellular DNA stability, it is also known to act as a general restriction factor for DNA viruses. To investigate the unique mechanism by which ATRX restricts HSV, we used a high-resolution sequencing-based assay to assess the accessibility of HSV DNA. We found that ATRX promotes reduced accessibility of viral DNA during infection, likely preventing access for factors necessary for viral gene expression and replication. Our study provides new insight into the mechanism by which ATRX acts as a generalized DNA virus restriction factor.

## Introduction

Epigenetic regulation is fundamental for the maintenance of appropriate basal cellular gene expression and to control expression changes in response to replication, stress, and invading pathogens. Eukaryotic DNA is wrapped around nucleosomes which allow for higher orders of compaction and the formation of larger chromatin domains [reviewed in [1]]. Nucleosomes are comprised of an octamer that contains 2 copies each of four core histone proteins, H2A, H2B, H3, and H4, around which are wrapped a 147 base pair (bp) segment of DNA [reviewed in [2]]. DNA-wrapped nucleosomes can assemble into a 10nm fiber and form higher order structures that enhance DNA compaction [3] and form regions of active or inactive gene expression. Post-translational modifications (PTMs), such as methylation, can be added to the tails of core histone proteins that comprise nucleosomes to recruit chromatin modifiers and further define regions of active and inactive gene expression [2].

As a DNA virus, herpes simplex virus (HSV) is subject to epigenetic regulation by host cell machinery. The double-stranded DNA (dsDNA) genome of HSV is not associated with histones in the virion [4,5]. However, several studies have shown that HSV genomes are rapidly loaded with histone H3 which is subsequently modified with heterochromatin-associated histone modifications such as histone H3 lysine 9 trimethylation (H3K9me3) and histone H3 lysine 27 trimethylation (H3K27me3) [4,6–9]. Which components of the cellular chromatin machinery are responsible for loading histones onto viral DNA upon nuclear entry is a question that remains to be answered.

Eukaryotic cells encode an array of histone chaperone proteins and complexes that facilitate the loading of histones onto DNA. Histone chaperones preferentially affect specific histone deposition pathways. This is partially governed by their affinities for particular histone variants that are expressed at different points of the cell cycle or are associated with specific activities, such as the role that phosphorylated histone H2AX plays in DNA damage signaling [10]. Histone H3 variants H3.1 and H3.2, for example, are only expressed during S phase while H3.3 is expressed throughout the cell cycle [11]. Therefore, histone variant-specificity of H3 chaperones can influence deposition activity. The chromatin assembly factor-1 (CAF-1) complex preferentially loads canonical H3.1/3.2 onto replicating DNA [11]. Conversely, the α-thalassemia X-linked intellectual disability (ATRX) and death domain-associated (DAXX) proteins form a complex that preferentially promotes H3.3 occupation at telomeres and pericentric repeats in a replication-independent process [12,13]. Another replication-independent H3.3 chaperone, HIRA, has a more generalized role in H3.3 loading, including histone loading at actively transcribed genes and a gap-filling chromatin maintenance function that can compensate for CAF-1 loss during cellular DNA replication [11,14].

Conflicting reports have precluded consensus on the roles that H3 chaperones play in viral chromatin formation and restriction [6,15–19]. HIRA and an upstream H3 chaperone, ASF1A, have been implicated in H3.3-biased occupation of histones on HSV DNA during lytic infection of HeLa cells [20,21]. Unexpectedly, these reports also suggested that HIRA and ASF1A promote viral gene expression and replication. Conflicting with these observations, it was recently reported that during HSV infection, HIRA localizes to promyelocytic leukemia nuclear bodies (PML-NBs) after interferon stimulation and promotes the expression of interferon-stimulated genes and contributes to the cellular antiviral response [17,18]. ATRX and DAXX have been well documented to inhibit numerous DNA viruses. These proteins have been shown to promote H3.3 occupation on the human cytomegalovirus (HCMV) [22], Epstein-Bar virus (EBV) [23], and adenovirus (AdV) [24] genomes at late times post infection. However, we recently showed that histone deposition on HSV DNA did not strictly require ATRX but that ATRX stabilized HSV chromatin during lytic infection and inhibited viral gene expression [6]. Highlighting the importance of this pathway in DNA virus restriction, HSV, HCMV, EBV, AdV, and Kaposi’s sarcoma associated herpesvirus (KSHV) all encode proteins that disrupt or degrade the ATRX/DAXX complex [19,24–28].

To further our understanding of the mechanism and machinery that govern chromatin formation during early lytic HSV infection, we investigated which H3 chaperones are responsible for *de novo* deposition of H3 to the HSV genome and what restrictive potential they might have during lytic infection. Using a series of combinatorial depletions of nuclear H3 chaperones, we found that only the ATRX/DAXX complex could significantly restrict replication of an ICP0-null herpes simplex virus. When transcription was inhibited globally, we found that only a combinatorial depletion of ATRX/HIRA/ASF1A resulted in a significant decrease in total H3 deposition by 4 h post infection (hpi), suggesting multiple pathways for chromatinizing naked DNA. Although not strictly required for *de novo* H3 deposition, ATRX was important for H3 retention on the viral genome, and ATAC-seq analysis revealed ATRX to be important for limiting HSV DNA accessibility before viral genome replication. The results presented here argue for a model in which *de novo* H3 deposition to incoming viral DNA occurs through multiple mechanisms but requires ATRX to restrict viral DNA accessibility, and effectively delay progression of the viral lifecycle.

## Results

### The ATRX/DAXX complex is more restrictive for ICP0-null HSV-1 replication than other nuclear H3 chaperone complexes

HSV encodes an E3 ubiquitin ligase, ICP0, that disrupts the restrictive effects of the ATRX/DAXX complex [19], in part due to the direct targeting the of the PML protein for proteasome-dependent degradation which results in the dissolution of PML-NBs [25]. ATRX has also been reported to be degraded sometime after PML in the presence of ICP0; however, there is currently no evidence that ATRX is a direct target of ICP0 [19,25]. Consistent with those results, we observed that the loss of ATRX in human foreskin fibroblast (HFF) cells infected with either an ICP0-null HSV-1 variant (7134) or the ICP0-rescued HSV-1 variant (7134R) requires ICP0 and can be detected by 6–8 hpi (S1A Fig). Using an EdC-labeled wild-type HSV-1 (KOS-EdC) [6] we were able to verify that in the presence of ICP0, PML-NBs are disrupted and that PML staining is lost by 2–3 hpi (S1B Fig). Unlike PML, ATRX staining was not abolished in the first 4 hpi; rather, it became diffuse throughout the nucleus of the infected cell. By 4 hpi, nearly 20% of infected cells displayed large ATRX aggregates (S1B and S1C Fig). Similar ATRX aggregates had been observed in GFP-tagged ATRX proteins with a mutation (D217A) in the H3K4me0 binding pocket [29]; however, ATRX aggregates had not been reported previously during HSV infection. Background staining in the HSV DNA panel in the uninfected (Mock) condition of S1B Fig was consistent with the streptavidin-GFP probe lacking a specific biotinylated target (biotinylated HSV DNA). During infection with HSV 7134, rather than forming aggregates, ATRX localizes to replication compartments, as marked by staining for HSV DNA binding protein ICP8, by 6 hpi (S1D Fig). Together, these results revealed a process by which ICP0 counteracts ATRX/DAXX complex restriction through the dissolution of PML-NBs and promoting the sequestration of ATRX away from viral DNA through aggregation. Because of the effects that ICP0 exerts on ATRX, we conducted the following experiments with HSV 7134 that is ICP0-null.

**Fig 1:**
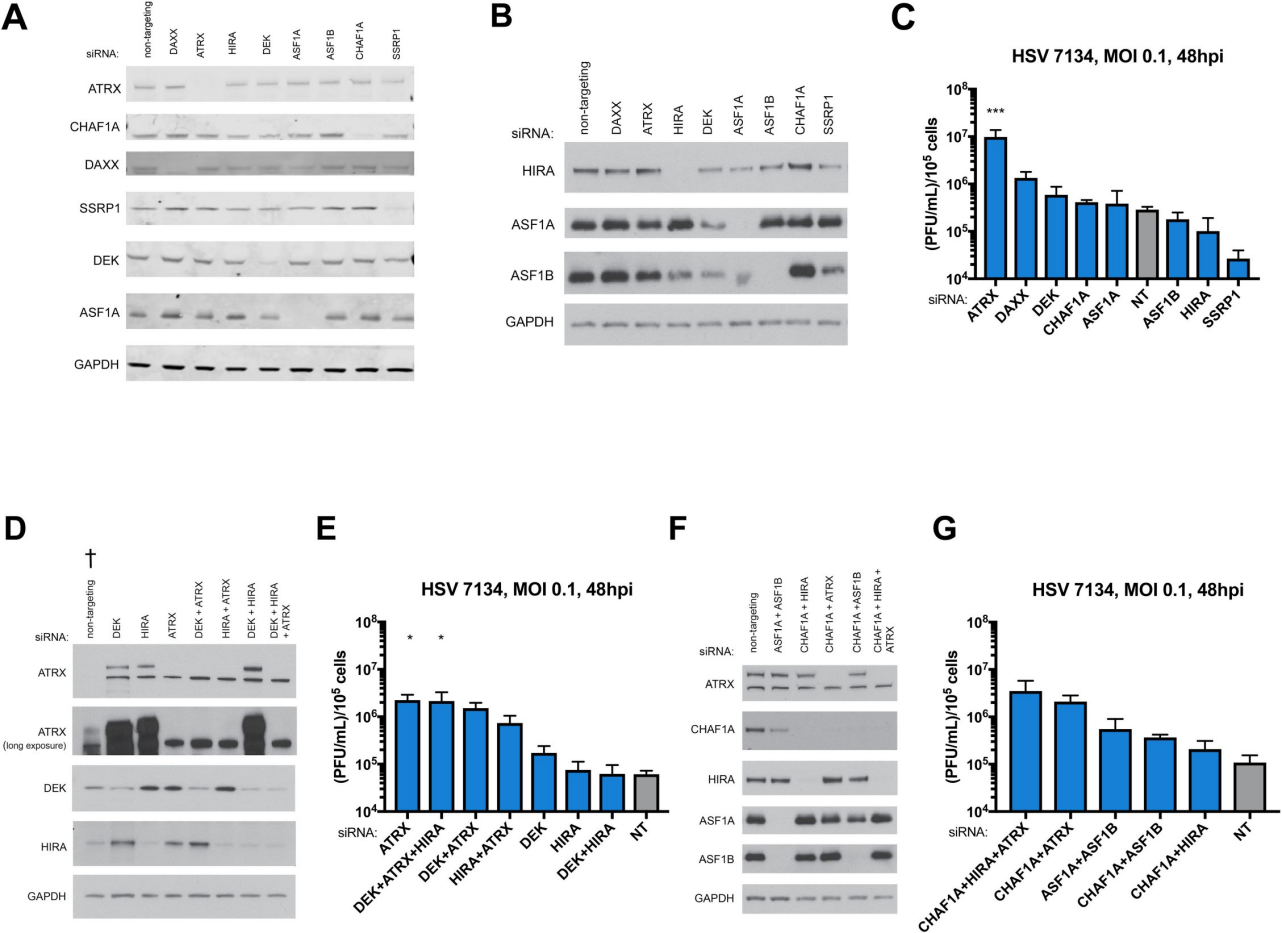
HSV yields after depletion of H3 chaperones by siRNA. (A) Immunoblot of lysates from TERT-HF cells treated with siRNA against nuclear H3 chaperones ATRX, CHAF1A, DAXX, SSRP1, DEK, ASF1A using a LiCor Odyssey imager. (B) Immunoblot of nuclear TERT-HF cells treated with siRNA against nuclear H3 chaperones HIRA, ASF1A, and ASF1B using x-ray film. Viral yields from TERT-HF cells treated with siRNAs (C) against non-targeting, ATRX, CHAF1A, DAXX, SSRP1, DEK, ASF1A, HIRA, and ASF1B. (D) Immunoblot of combinatorial siRNA treatments against ATRX, DEK, and HIRA using x-ray film († Mechanical failure of a freezer led to partial degradation of the siNon-Targeting treated sample). (E) Viral yields from TERT-HF cells treated with combinatorial siRNA treatments against ATRX, DEK, and HIRA. (F) Immunoblot of combinatorial siRNA treatments against ATRX, CHAF1A, HIRA, ASF1A, and ASF1B using x-ray film. (G) Viral yields from TERT-HF cells treated with combinatorial siRNA treatments against CHAF1A, HIRA, ATRX, ASF1A, and ASF1B which were infected with HSV 7134 at an MOI of 0.1. Viral lysates were collected at 48 hpi and titrated on U2OS cells. Yields were normalized to (PFU/mL)/1x10^5^ cells. Results were analyzed by One-way ANOVA using Dunnet’s multiple comparison correction. Data are reported as the average of 3 independent experiments ± standard error of the mean; p < 0.05 (*), p < 0.01 (**), p < 0.001 (***).

Recently, we demonstrated that despite its ability to restrict HSV gene expression and replication, the ATRX/DAXX complex is not strictly required for the initial deposition of H3 on incoming ICP0-null HSV genomes [6]. However, a thorough analysis of whether other nuclear H3 chaperones also have the capacity to restrict HSV replication has yet to be performed. To determine if other nuclear H3 chaperone complexes could also restrict ICP0-null HSV replication, we used siRNAs to systematically deplete hTERT-immortalized human foreskin fibroblasts (TERT-HF) of various histone H3 chaperone components (Fig 1A and 1B). At 72 h after siRNA treatment, we infected cells with 7134 virus at an MOI of 0.1 and determined viral titers at 48 hpi. Interestingly, ATRX was the only H3 chaperone whose depletion resulted in a statistically significant increase in HSV yields (Fig 1C).

It has been reported that the ATRX-binding domain of DAXX is required for DAXX-mediated restriction of HSV [19], and we recently showed that depletion of DAXX in ATRX-KO fibroblasts is not additive [6]. Thus, the restrictive capacities of ATRX and DAXX are likely a function of the ATRX/DAXX complex and not the individual subunits. Although not statistically significant using the stringent Bonferroni correction for multiple comparisons, depletion of DAXX, the binding partner of ATRX, increased viral yield by approximately 5-fold (Fig 1C). DAXX depletion has consistently resulted in comparatively smaller increases in viral yield than ATRX depletion (Fig 1C) [6], and DAXX has recently been reported to have potential roles in viral activities during late infection [6,30]. We previously observed no additive effect of DAXX and ATRX co-depletion [6]. For these reasons, we chose to focus on ATRX depletion to investigate the role of the ATRX/DAXX complex.

HIRA is another nuclear H3.3 specific chaperone complex that has been implicated in loading H3.3 to HSV genomes [20]. Depletion of HIRA resulted in minor non-significant changes in viral yields that were both slightly above and slightly below siNT treatment depending on the experimental series (Fig 1C–1E). Depletion of CHAF1A, a component of the replication-dependent H3.1/3.2 chaperone complex CAF-1, also resulted in non-significant changes in viral yield (Fig 1C). Likewise, depletion of ASF1A and ASF1B, H3 chaperones that are thought to function in the HIRA and ATRX/DAXX pathways, resulted in non-significant changes in viral yield (Fig 1C). It should be noted that siRNA depletion of ASF1A also appears to have resulted in a depletion of ASF1B (Fig 1A and 1B). Treatment with siASF1B did not result in decreased ASF1A (Fig 1A and 1B). However, a double depletion of ASF1A and ASF1B led to a small increase in viral yield compared to ASF1A or ASF1B alone (Fig 1C and 1G) suggesting that the depletion of ASF1B as a result of siASF1A treatment was partial. Surprisingly, depletion of SSRP1, a component of the transcriptionally coupled FACT complex, resulted in moderate, but non-significant decreased viral yields (Fig 1C). This observation, combined with a recent report that components of the FACT complex including SSRP1 associate with input viral DNA throughout the first 6 h of infection [31], suggested that the FACT complex may play a role in promoting viral gene expression. The same study also observed that DEK, a H3.3 chaperone that regulates H3.3 distribution to HIRA and ATRX/DAXX [32], associated with the viral genome for most of the first 6 h of infection. Depletion of DEK resulted in a 2–3 fold increase in viral yield (Fig 1C and 1E), the largest increase in viral yield outside of DAXX and ATRX. DEK may possess a minor capacity to restrict ICP0-null HSV. It is also possible that DEK may be inhibited by another HSV factor. However, it should be noted that treatment with siDEK appears to also result in a minor depletion of ASF1A and ASF1B (Fig 1A and 1B)

Combinatorial depletions of nuclear H3 chaperones yielded large increases in viral yield only when ATRX was also depleted (Fig 1D–1G). While experimental variation and stringent multiple correction criteria preclude statistical significance, it is worth noting that the CHAF1A+ATRX and CHAF1A+HIRA+ATRX ranged from 11-38-fold and 17–40 fold higher yield than treatment with siNT, respectively (Fig 1G). Western blot analysis showed that all combinations of siRNAs led to protein depletions as well as single siRNAs (Fig 1A, 1B, 1D and 1F). Blots for ATRX developed with ECL substrate showed two bands with the upper representing ATRX (Fig 1D and 1F). While there is always the possibility that the siRNA depletions of some H3 chaperones was not sufficient to reveal restrictive properties, the major effect of ATRX depletion on viral yields suggested that the ATRX/DAXX complex exhibits a potent restrictive function to limit lytic ICP0-null HSV infection that is not shared by other nuclear H3 chaperones.

Recently, we demonstrated that ATRX is required to maintain viral chromatin stability during chromatin stress, such as during transcription and DNA replication [6], but it is not known if other H3.3 chaperones also contribute to viral chromatin stability. We therefore performed chromatin immunoprecipitation-quantitative polymerase chain reaction (ChIP-qPCR) analysis of chromatin from cells depleted for 3 chaperones with siRNA. In support of the unique role that ATRX plays in restricting ICP0-null HSV infection, we found that during combinatorial co-depletion of HIRA, DEK, and ATRX, total H3 was decreased only in cells depleted of ATRX (S2A and S2B Fig). Similarly, viral genomes were elevated in ATRX-depleted cells when compared to siNT, but siDEK, and siHIRA treated cells were not (S2C Fig). As observed previously [6], ATRX depletion increased ICP27 and gB mRNAs significantly, but ICP8 mRNA was increased to a lesser extent (S2D Fig). Individual depletion of DEK or HIRA did not greatly enhance viral transcript levels from immediate-early (IE), early (E), and late (L) viral genes, as represented by those from the *ICP27*, *ICP8* and *gB*, respectively, even in the presence of acyclovir, a viral polymerase-specific inhibitor (S2D and S2E Fig). Co-depletion of HIRA and DEK resulted in a significant increase in *ICP8* expression and minor but significant increase in *gB* gene expression (S2D Fig). These results may indicate that together DEK and HIRA play a role in the regulatory mechanisms that govern viral gene expression or the kinetics of the viral gene cascade. However, despite the changes in viral gene expression, co-depletion of DEK and HIRA resulted in neither elevated viral yield (Fig 1D) nor increased accumulation of viral DNA at 8 hpi (S2C Fig). This suggested that the observed changes in viral gene expression during DEK and HIRA co-depletion do not impact viral replication. These results supported the observation that the ATRX/DAXX complex has an enhanced capacity to affect viral chromatin stability and to restrict ICP0-null HSV as compared to other H3.3 chaperones.

### Multiple H3 chaperone pathways contribute to the loading of H3 to incoming HSV DNA

**Fig 2:**
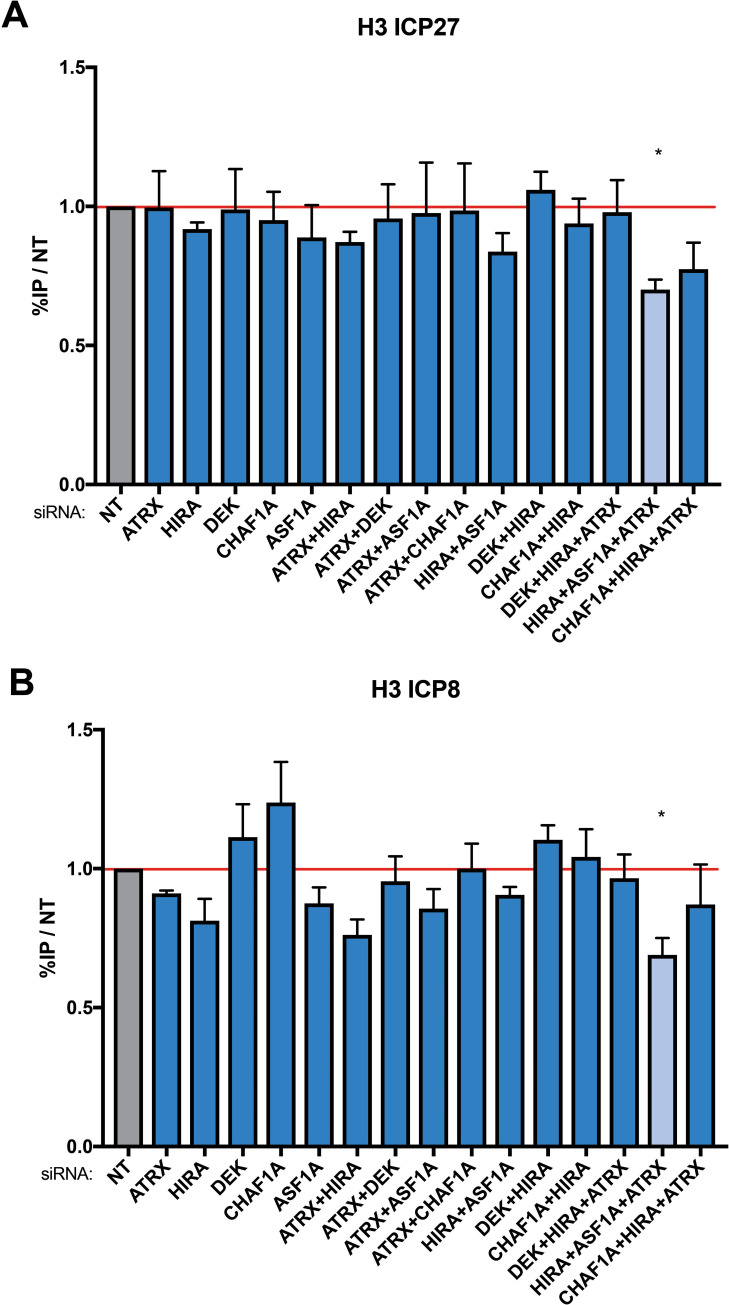
Detection of H3 on promoters of HSV genes *ICP27* and *ICP8* by ChIP-qPCR after H3 chaperone depletion. TERT-HF cells were treated with siRNAs against non-targeting (NT), ATRX, HIRA, DAXX, CHAF1A, DEK, and ASF1A. 72 h post siRNA treatment, cells were infected with HSV 7134 at an MOI of 3 in the presence of flavopiridol. Infected cells were fixed and harvested 8 hpi. ChIP-qPCR using a pan-H3 antibody and HSV specific primers were used to detect enrichment of H3 at viral gene promoters for (A) ICP27 and (B) ICP8. Results reported as Relative IP (the percent of input immunoprecipitated by each antibody normalized to the 8-hour control sample—set to 1.0 for each replicate). Results were analyzed by one sample T-test. Data are reported as the average of 3 independent experiments ± standard error of the mean; p < 0.05 (*), p < 0.01 (**), p < 0.001 (***).

We recently observed that the restriction of HSV by the ATRX/DAXX is not mediated through the initial loading of H3 to the viral DNA [6]. However, some histone chaperones can compensate for the loss of other H3 chaperones [14,33]. It is currently unknown which H3 chaperones are responsible for *de novo* loading of H3 to incoming HSV DNA. To determine if the initial deposition of H3 to the input viral DNA occurs through a single chaperone or a set of H3 chaperone pathways, we used siRNAs to deplete H3.3 and H3.1 chaperones individually or in combination in TERT-HFs. Because transcription disturbs chromatin stability [34], we used the CDK9 inhibitor flavopiridol to inhibit transcription during infection to determine which H3 chaperones might be responsible for *de novo* H3 deposition rather than maintenance. At 4 hpi with 7134 virus, single depletion of H3 chaperones did not result in significant changes in H3 occupation at the *ICP27* or *ICP8* gene promoters (Fig 2A and 2B). Similarly, double depletion of H3 chaperones resulted in minor or no changes in viral H3. However, triple depletion of HIRA/ASF1A/ATRX consistently resulted in an approximate 30% reduction of viral H3 at both the *ICP27* and *ICP8* gene promoters (Fig 2A and 2B). This demonstrated that the initial deposition of H3 to incoming viral DNA is not dependent on individual nuclear H3 chaperones. Rather, it suggested that H3 is deposited through a set of H3 chaperones that can compensate for the loss of an individual chaperone complex or through non-canonical pathways such as the one suggested to be mediated by the NAP1-Like proteins [35,36].

### ATRX-mediated restriction of HSV does not require H3K9me3 histone lysine methyltransferases SUV39H1 and SETDB1 or HDAC recruitment

The ATRX/DAXX complex has been shown to promote H3K9me3 at telomeres and to interact with histone methyltransferases (HMTs) SUV39H1 and SETDB1 [37,38]. It is widely thought that H3K9me3 modification to viral H3 would act to restrict viral gene expression and replication. ATRX co-depletion with SETDB1 and/or SUV39H1 (S3A Fig) resulted in minor decreases in viral yield compared to ATRX-alone (S3B Fig) demonstrating that co-depletion of ATRX and these HMTs was not additive. It is important to note that siRNA depletion of SETDB1 was only partial (S3A Fig) and may have not been sufficient to reveal restrictive properties. Single depletion of SUV39H1 in HFFs or treatment of ATRX-knockout (ATRX-KO) and Cas-9-only control (Control) cells [6] with chaetocin (10nM), an inhibitor of SUV39H1, resulted in reduced viral yield (S3B and S3C Fig). Overall, these results argued that these HMTs were not strictly required for the ATRX/DAXX complex-mediated restriction of HSV.

It was possible that the ATRX/DAXX complex restricts DNA viruses through the recruitment of histone deacetylases (HDAC) [26,39,40]; therefore, we infected ATRX-KO and Control cells treated with and without an HDAC inhibitor, trichostatin A (TSA) (100ng/mL). Viral yields were reduced similarly in ATRX-KO and Control cells treated with TSA when compared to cells treated with vehicle only (S4A Fig). The reduction of viral yield during TSA treatment was surprising and conflicts with a previous report that TSA treatment can enhance ICP0-null HSV gene expression [41]. However, a more recent study described a reduction in the number of HSV genomes initiating expression in TSA-treated cells [42]. These findings argued that the ATRX/DAXX complex does not restrict HSV through the recruitment of HDACs.

### Loss of ATRX enhances viral gene expression but has little effect on cellular gene expression

**Fig 3:**
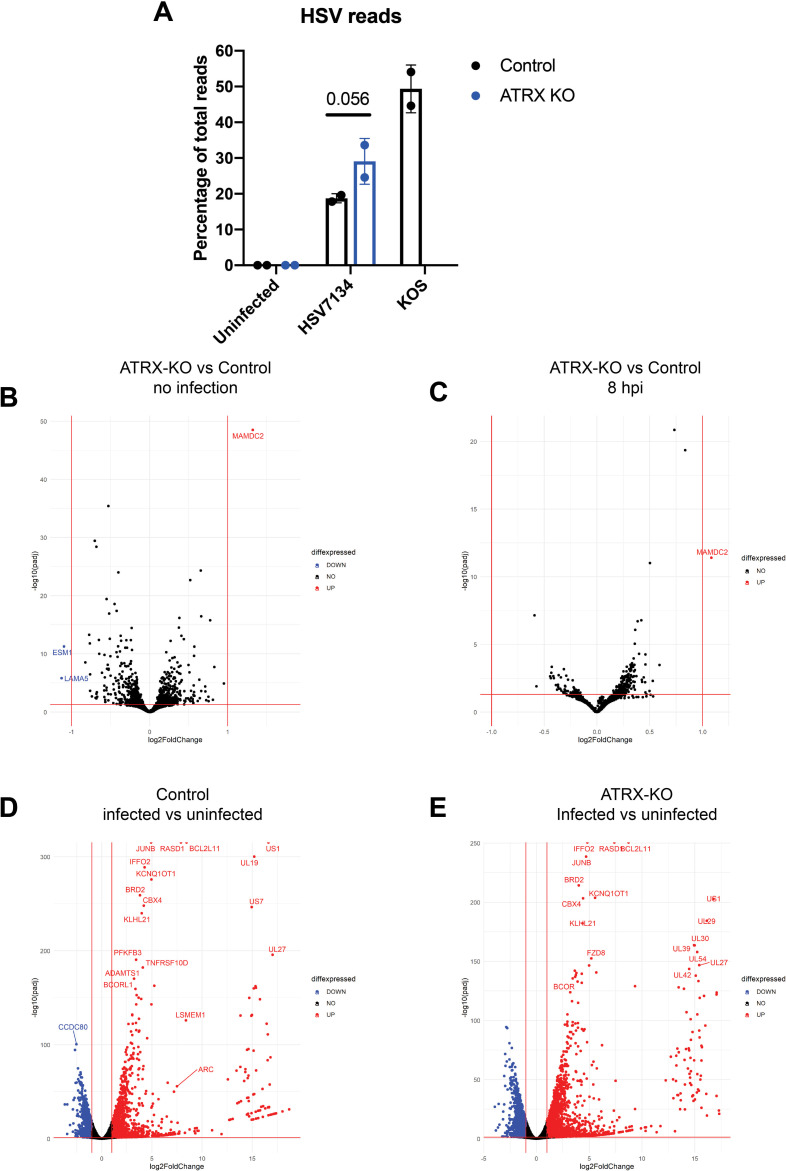
RNA-seq analysis of viral and human transcripts during HSV infection. (A) Reads mapped to the HSV genome as a percentage of total mapped reads (HSV+human) per sample. Data shown are the average of 2 independent experiments. Differential gene expression of human and HSV genes in (B) uninfected ATRX-KO cells/uninfected Control cells, (C) 7134 infected ATRX-KO cells/7134 infected Control cells at 8 hpi, (D) 7134 infected Control cells 8 hpi/uninfected Control cells, (E) 7134 infected ATRX-KO cells at 8 hpi/uninfected ATRX-KO cells as determined by comparing fold change vs adjusted p-value (padj). Data shown is the average of 2 independent experiments.

We next investigated if loss of ATRX induced changes in cellular gene expression that could account for the ATRX-mediated restriction of HSV. Using ATRX-KO and Control cells, we performed RNA-sequencing (RNA-seq) analysis of poly(A)-enriched RNA harvested from infected and uninfected cells. We infected cells that only express Cas9 (Control) with wild-type HSV (WT) as a control. Across the viral genome, the expression pattern of HSV-1 genes observed from WT infected cells at 8 hpi (S5) closely resembled the gene expression pattern described for HSV-1 at this time point [43]. Total HSV gene expression was enhanced in ATRX-KO cells (Figs 3A and S5). At 8 hpi, HSV reads constituted 18.7%, 29.1%, and 49.3% of all mapped reads (human + HSV) in Control cells infected with 7134, ATRX-KO cells infected with 7134, and Control cells infected with KOS, respectively (Fig 3A). These results revealed that ATRX affects ICP0-null HSV gene expression with depletion of ATRX leading to an overall increase in viral transcripts.

We next investigated the effects that ATRX depletion has on cellular gene expression. Surprisingly, cellular gene expression in ATRX-KO cells was remarkably similar to Control cells in the absence of HSV infection (Fig 3B). No known HSV restriction factors were relatively down regulated in ATRX-KO cells (S1 Table). MAMDC2 was the only gene identified as upregulated in our analysis. An antisense transcript, MAMDC2-AS1, from this locus was recently described as upregulated during HSV infection and was described to promote infection through enhancing the nuclear transport of a viral transactivator, VP16 [44]. However, high level of MAMDC2-AS1 overexpression from a transfected vector had a smaller than two-fold effect on viral DNA replication and plaque forming efficiency. MAMDC2-AS1 was not significantly upregulated in ATRX-KO cells before or during infection as compared to Control cells (S6C and S6D Fig and S2 Table). Thus, the 2-fold up regulation of MAMDC2 seems unlikely to account for the changes in viral replication observed during ATRX depletion.

At 8 hpi, the cellular gene expression of Control and ATRX-KO cells was also very similar (Fig 3C) and included relative upregulation of several stress response related genes including heat shock-protein 70 (HSPA1B) and H2AX (H2AFX) (Fig 3D and 3E and S1 Table). Of genes identified as down-regulated during infection, 92% of downregulated genes in Control cells were also downregulated in ATRX-KO cells (S6A Fig). Similarly, 90% of upregulated genes in Control cells were also upregulated in ATRX-KO cells (S6B Fig). The enhanced kinetics of HSV infection in ATRX-KO cells may account for the increased number of up- and downregulated genes as HSV infection has dramatic effects on the cellular transcriptome [45–48]. The similarities in cellular gene expression between ATRX-KO and Control cells both before and during HSV infection argued that the ATRX/DAXX complex-mediated restriction of HSV was likely the result of a direct effect rather than an indirect or off-target effect due to ATRX-depletion.

### ATRX limits HSV genome accessibility during early lytic infection

**Fig 4:**
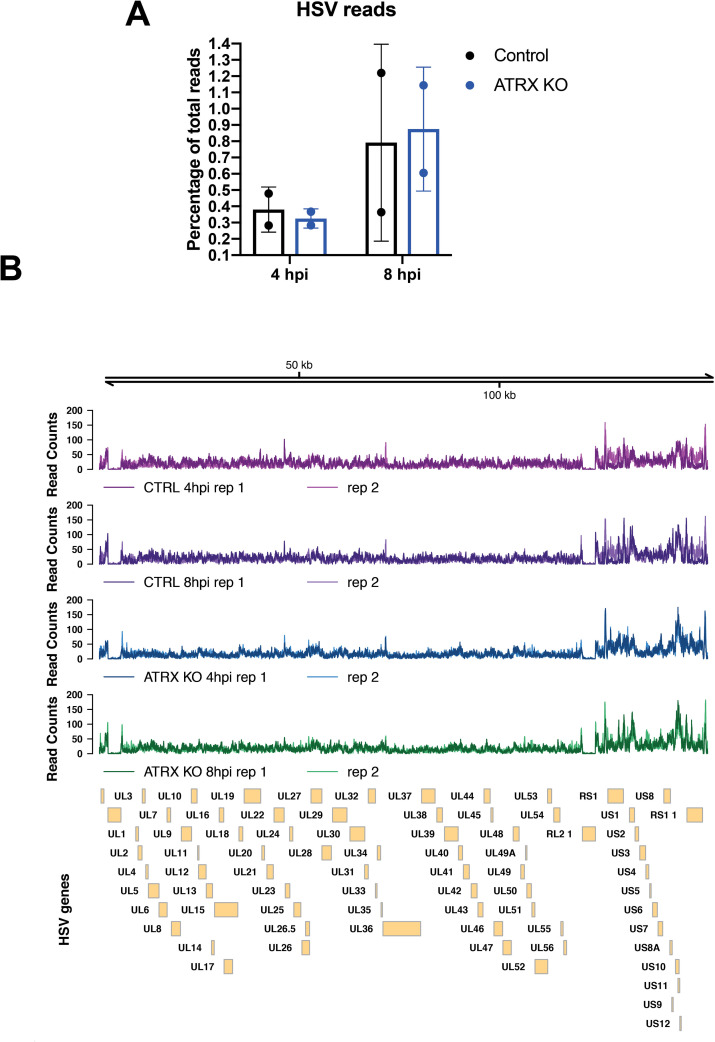
Levels of H3 on HSV genome in infected cells at 4 and 8 hpi by ChIP-seq. H3 ChIP-seq reads in ATRX-KO or Control cells infected with 7134 at MOI3 and harvested at 4 or 8 hpi were aligned to a human+HSV genome. (A) The percentage of HSV reads to total aligned reads was calculated. (B) Alignments were visualized across HSV genome after normalizing reads to total HSV reads. Data shown in Fig 4 is from 2 independent experiments.

Chromatin immunoprecipitation sequencing (ChIP-seq) was used to assess the distribution of H3 across the entire HSV genome. High levels of histone H3 were detected on the HSV genome by 4 hpi in both ATRX-KO and Control cells (Figs 4A and 4B and S7). Alignment of the ChIP-seq reads revealed a dense distribution of H3 across the entire HSV genome in both ATRX-KO and Controls during infection (Fig 4B). Samples were normalized to total HSV reads to view read distribution patterns on the same scale. Analysis of H3 distribution detected no differential peaks between Control and ATRX-KO cells (Fig 4B). Therefore, the quantity and distribution of histone H3 was similar with or without ATRX across the entire HSV genome, significantly extending our previous ChIP-qPCR studies on a limited number of sites on the viral genome [6].

**Fig 5:**
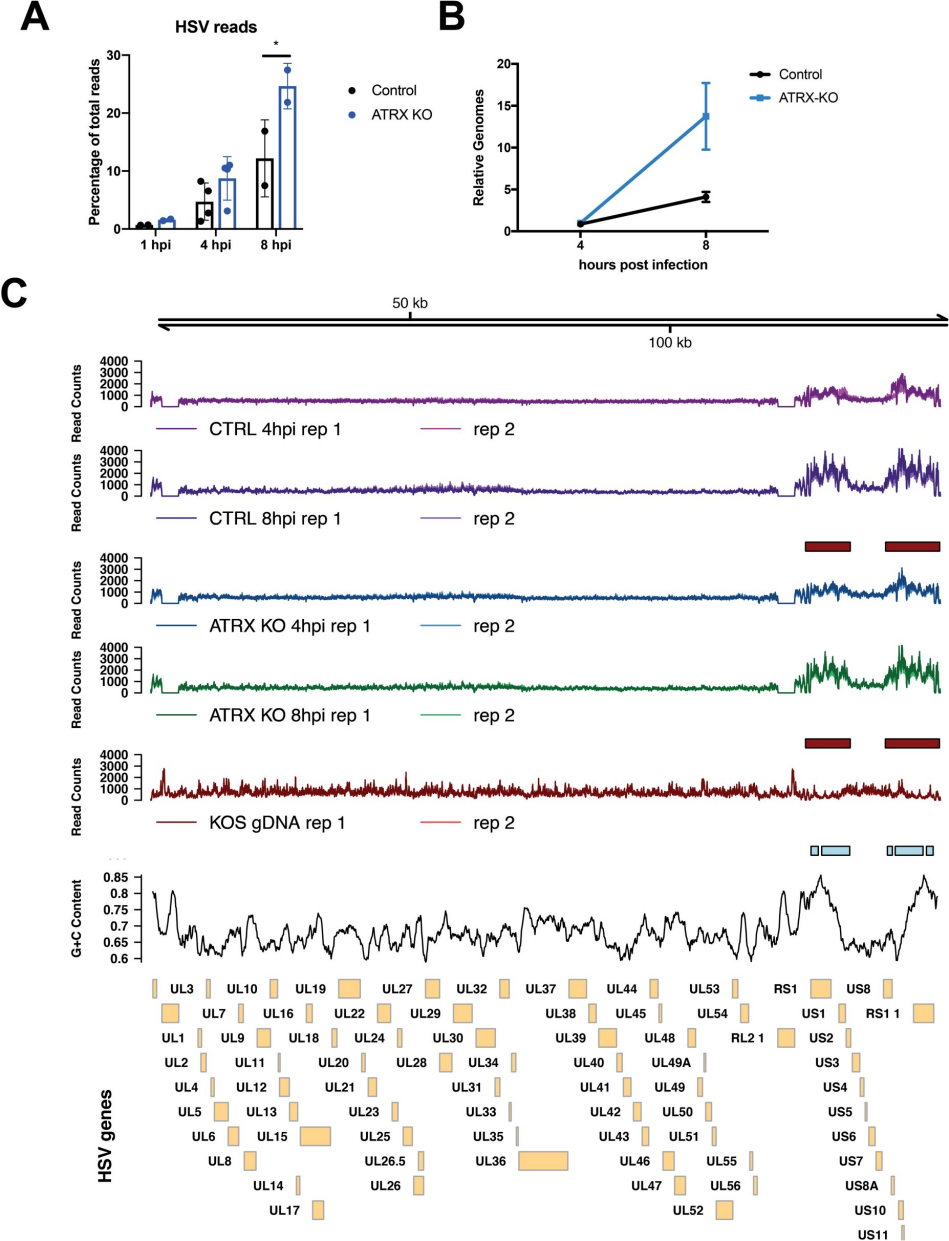
Accessibility of HSV DNA during infection as determined by ATAC-seq. ATAC-seq was used to determine accessibility of the HSV genome in ATRX-KO or Control cells that were infected with an ICP0- HSV (7134) at MOI 5 and harvested at 1, 4, and 8 hpi. (A) Reads were aligned to a human+HSV genome and percentage of HSV reads to total mapped reads was calculated. (B) Relative HSV genomes were quantified by qPCR analysis of ICP8/GAPDH signals in TN5 treated ATRX-KO and Control samples at 4 and 8 hpi with 7134. Data are reported as the average of 2 independent experiments, except for the 4hpi data in (A) in which 4 replicates were included, ± standard error of the mean; p < 0.05 (*). (C) ATAC- seq reads that align to the HSV genome in samples from proteinase K treated HSV (KOS) genomes, Control cells and ATRX-KO cells infected with 7134 at MOI 5 and harvested at 4 and 8 hpi. Reads were normalized to total HSV reads prior to visualization. Boxes underneath tracks indicate regions of significant (FDR < 0.05) differential accessibility (red = increased accessibility, blue = decreased accessibility) between ATRX-KO cells infected with 7134 at 8 hpi vs 4 hpi, Control cells infected with 7134 at 8 hpi vs 4 hpi, and proteinase K-treated KOS genomes vs Control cells at 8 hpi. GC content in 1000 bp windows across HSV KOS genome is visualized for comparison.

We next investigated the effects of the ATRX/DAXX complex on HSV DNA accessibility in viral chromatin with the Assay for Transposase-Accessible Chromatin using sequencing (ATAC-seq) technique [49]. ATRX has been shown to play a major role in chromatin stability during replication and DNA repair [15,16,50–52], and ATRX has also been reported to affect chromatin susceptibility to micrococcal nuclease (MNase) digestion [53]. To test if ATRX likewise affects HSV DNA accessibility, we infected Control and ATRX-KO cells with 7134 virus and performed ATAC-seq at 1, 4, and 8 hpi. HSV genome reads made up a larger percentage of total ATAC-seq reads in ATRX-KO cells at 1, 4, and 8 hpi (Fig 5A), before and after the start of viral DNA replication (Fig 5B). Despite having similar viral genome copy numbers during the first 4 hpi (Fig 5B), HSV DNA accounted for 0.69% and 1.6% of total mapped reads (HSV+Human) at 1 hpi in Control and ATRX-KO cells, respectively. By 4 hpi, HSV DNA accounted for 8.7% of all mapped reads in ATRX-KO cells and only 4.7% in Control cells (Fig 5A). HSV ATAC-seq reads continued to increase as a percentage of total mapped reads, and by 8 hpi, these numbers increased to 24.7.75% and 12.2% in ATRX-KO and Control cells, respectively. A similar overrepresentation of HSV in ATAC-seq reads was recently reported during WT infection [54], demonstrating that HSV genomes are far more accessible than cellular chromatin during early infection. These results argued that ATRX mediates HSV DNA accessibility during lytic infection.

While the percentage of reads mapping to the HSV genome was higher at 4 hpi in ATRX-KO vs Control cells (Fig 5A), the distribution pattern was remarkably similar when the samples were normalized by total HSV reads (Fig 5C). The ATAC-seq reads were distributed across the entire HSV genome indicating a high level of accessibility genome wide (Fig 5C). ATAC-seq was also performed on WT viral DNA (KOS gDNA) that was purified on a sodium iodide gradient and treated with proteinase K. Little or no DNA read coverage was observed at either ICP0 gene locus in 7134 infected cells, whereas the purified WT HSV DNA mapped reads at the ICP0 genes, validating the HSV genome alignment (Fig 5C). The viral chromatin showed increased accessibility in the unique short (US) region of the HSV genome and the flanking repeat regions in both the infected ATRX-KO and Control cells as compared to the purified genomic HSV DNA (KOS) (Fig 5C). Differential peak analysis found no differences between the normalized reads of the ATRX-KO and Control conditions at either time point, but it did show significant increase in accessibility in the HSV US and flanking repeat regions from 4 to 8 hpi in both ATRX-KO and Control Cells (Fig 5C). The accessibility of the purified KOS gDNA was significantly lower than in Control cells at 8 hpi through these same regions (Fig 5C). The regions of enhanced accessibility during infection overlapped with areas of elevated viral gene expression as observed during RNA-seq analysis (S5 Fig) as well as to areas of enriched GC content (Fig 5C). In ATRX-KO and Control cells, we identified a weak positive correlation between normalized ATAC-seq counts and GC content through the HSV US and flanking repeat regions (S8A, S8B and S8D Fig). In contrast, we identified a stronger negative correlation between normalized ATAC-seq counts and GC content in the purified WT HSV DNA samples (S8C and S8D Fig).

**Fig 6:**
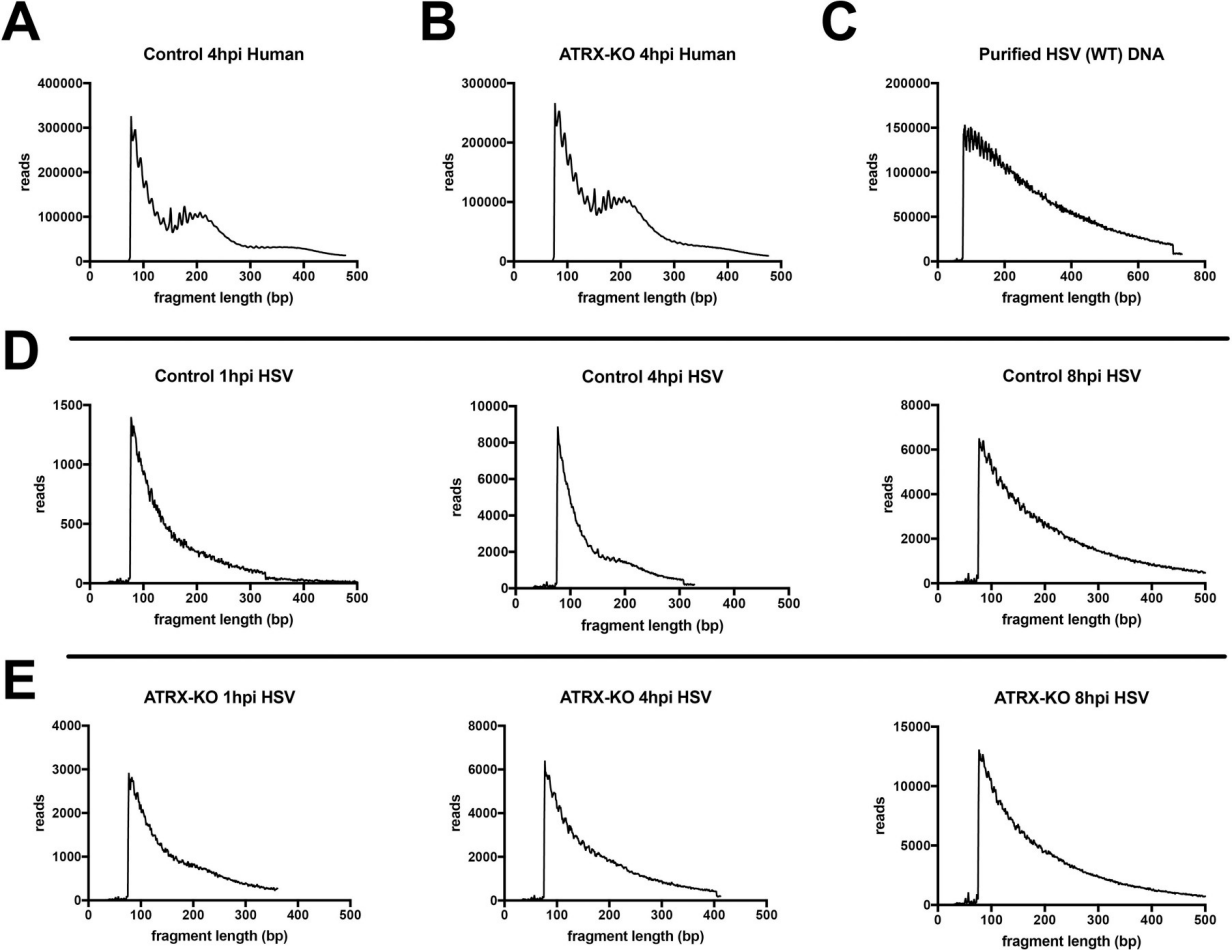
Frequency distribution of ATAC-seq DNA read lengths during HSV infection. The frequency distribution of sequenced fragment lengths of reads that aligned to the human genome in Control (A) and ATRX-KO (B) cells infected with an ICP0- HSV at MOI 5 and harvested at 4 hpi. (C) The frequency distribution of sequenced fragment lengths of reads that aligned to the HSV genome in proteinase K treated, purified HSV (WT) DNA. The frequency distribution of sequenced fragment lengths of reads that aligned to the HSV genome in Control (D) and ATRX-KO (E) cells infected with an ICP0- HSV at MOI 5 and harvested at 1, 4, and 8 hpi. Data shown in Fig 6 is representative of 2 independent experiments.

Examination of the fragment length distribution of human DNA reads in both ATRX-KO and Control cells revealed a bimodal distribution of fragment lengths that mapped to the human genome, with peaks at 200 bp and 400 bp that indicated enrichment for DNA fragments corresponding to mono- and di-nucleosome protected DNA (Fig 6A and 6B). In contrast, purified, treatment of protein-depleted WT HSV DNA resulted in a right-skewed distribution of sequenced fragment lengths without enrichment of 200 or 400 bp fragment lengths (Fig 6C). However, the 4 hpi Control sample displayed a slight enrichment of the sequenced fragment length histogram at around 200 bp (Fig 6D). This enrichment is also observed in a second replicate, although the enrichment at 200bp is less pronounced (S9B Fig). There may also be a slight enrichment of 200bp fragments that map to the HSV genome in the ATRX-KO 1hpi samples (Figs 6E and S9A). Like protein-depleted WT HSV DNA, the distribution of HSV fragment lengths in ATRX-KO cells at 4 hpi was not bimodal and did not show enrichment for 200 or 400 bp fragment lengths (Fig 6E). These results suggested that, in the presence of ATRX, a subpopulation of HSV DNA fragments were protected from Tn5 transposase activity in nucleosome-sized fragments of DNA 4 hpi.

## Discussion

Preventing the expression of foreign gene products is paramount in the cellular effort to defend itself against intracellular pathogens. Once viral proteins are expressed, they disrupt normal cellular function and hijack cellular pathways for the purpose of viral propagation, a process that often results in injury or death of the infected cell. To prevent such hostile takeovers, cells mobilize factors, including chromatin-modifying proteins, to restrict gene expression and replication of invading DNA viruses. In this study, we demonstrate that the ATRX/DAXX complex has a higher capacity to restrict ICP0-null HSV gene expression and replication than do other histone H3 chaperones. The *de novo* deposition of H3 onto viral genomes appears to happen through multiple H3 chaperone pathways that can compensate for the loss of individual chaperones complexes, and histone H3 does not largely organize into fully formed nucleosomes on the HSV genome during lytic infection. The ATRX/DAXX complex limits accessibility of HSV DNA to Tn5 transpose activity during ATAC-seq, suggesting a possible mechanism for viral restriction. These observations support a model in which ATRX restricts invading DNA virus replication by altering the structure of histone H3-loaded viral chromatin so as to limit viral DNA accessibility. We concluded previously [6] that ATRX promotes the maintenance of viral heterochromatin, while our new results argue instead that ATRX alters the structure of viral chromatin and this results in reduced accessibility and increased retention.

### Viral histone loading through multiple chaperone pathways

Histone chaperones fulfill specific functions in the establishment and maintenance of cellular chromatin, but the roles that they play in true *de novo* chromatin formation in living cells are not well understood. HSV infection has proven to be a uniquely suited tool for investigating this process. In the absence of transcription, we found that no single nuclear H3 chaperone was required for *de novo* deposition of histone H3 on viral DNA. Even dual depletions of H3 chaperones had only minor effects on H3 occupation at HSV gene promoters. Only triple depletion of HIRA, ATRX, and ASF1A, components of the three major, nuclear H3.3 specific chaperone pathways, resulted in a significant reduction in H3 loading by 4 hpi. This argues that multiple pathways can deposit H3 on naked DNA *de novo*, and that these pathways can compensate for defects in any individual complex in terms of overall H3 loading, although the composition of H3 variants may be altered. It is tempting to speculate that this may also be a model by which *de novo* chromatin may form on any nucleosome-free piece of nuclear DNA.

The state of chromatin at a locus is often informed by the chromatin features of surrounding regions. Newly replicated eukaryotic DNA is loaded with parental histones which are used to re-establish the parental epigenetic state [reviewed in [55]]. Recycling of parental histones can reproduce positional information of histone post-translational modifications (PTMs) on newly synthesized DNA [56]. Nuclear naked DNA is rarely encountered by cells outside of the context of cell death or viral infection. Thus, naked DNA in the nucleus represents a danger signal to cells and may elicit an “all-hands-on-deck” response to which any histone chaperone capable of loading histones can be recruited. It is also possible that proteins, such as NAP1L1, can load H3-containing nucleosome-like structures as has been observed *in vitro* [35]. Further studies will be necessary to fully map the required proteins and pathways for *de novo* histone deposition; however, this study suggests that *de novo* histone loading to naked DNA may be more chaperone agnostic than previously believed. Our results support a model in which H3 may be loaded by multiple chaperone pathways and that maintenance or stabilization may be specific to particular H3 chaperones depending on the chromatin context. A recent paper on the role that ATRX plays in DNA damage provides additional support for this hypothesis. It was reported that HIRA, but not ATRX was required for H3.3 loading onto sites of double strand break repair at early times during the repair [16]. However, at later times post infection, ATRX was required for H3.3 occupation at the same site with no requirement for HIRA. As the context of the chromatin changed, so did the histone chaperone complex required for maintenance. HSV will continue to be a unique and high-resolution tool with which to investigate these pathways.

Previous studies have reported that HIRA and ASF1A promote H3.3 and total H3 occupation, respectively, on the HSV genome during early lytic infection in HeLa cells [20,21]. Here we show that neither HIRA nor ASF1A are strictly required for *de novo* loading of H3 onto the HSV genome in HFF cells. This discrepancy may be accounted for by the fact that the HIRA and ASF1A studies were performed in rapid cycling transformed HeLa cells that have altered chromatin stability, as evidenced by their hyperploidy. Another plausible explanation might be a difference in HSV strains. While those studies used an ICP0-positive wild-type virus, here we employ an ICP0-null HSV to investigate cellular mechanisms that might be disrupted by the activity of ICP0. Because the activity of the ATRX/DAXX complex is disrupted by ICP0, depletion of other histone chaperones during WT HSV infection is effectively a double depletion. We found that a triple depletion ASF1A/ATRX/HIRA was necessary for significant reduction in total *de novo* H3 loaded to viral DNA in the absence of transcription. Thus, depletion of ATRX and other restriction factors by ICP0 combined with depletion of another H3 chaperone may be sufficient to reduce H3 on the HSV genome.

### Restriction of HSV is likely mediated by a direct effect of ATRX

The ATRX/DAXX complex is the only nuclear H3 chaperone complex that exhibited the capacity to significantly restrict ICP0-null HSV in this study. Like the other H3 chaperones, the ATRX/DAXX complex is not strictly required for H3 deposition to the HSV genome in the absence of transcription [6]. We recently proposed a model in which epigenetic restriction of HSV occurs in a biphasic manner: H3 is loaded on the HSV genome, and then a second maintenance phase, mediated by ATRX, acts to restrict the virus. Our current results argue instead that ATRX promotes a structure of H3-loaded viral chromatin that is less accessible to nucleases and transcription.

A corollary of this is that H3 may not be inherently restrictive and may require the activity of additional host factors to yield viral chromatin that restricts viral gene expression and replication.

The ATRX/DAXX complex has numerous functions in regulating cellular processes that extend beyond its function as a histone chaperone. The ATRX/DAXX complex is known to recruit both HDACs and HMTs to modulate histone PTMs and regulate chromatin stability and gene expression [37,40,53]. However, when we perturbed several of these pathways, we could find no difference in the effects on ATRX-KO versus Control cells. The remarkably similar human gene expression profiles between Control and ATRX-KO cells argue that this restriction is likely a direct rather than an indirect effect of ATRX depletion. This argument is supported by the numerous proteins encoded by a variety of DNA viruses that disrupt or manipulate the ATRX/DAXX complex. HSV, HCMV, and AdV encode viral proteins that drive the degradation of either ATRX or DAXX [24–26] while EBV and human papilloma viruses (HPVs) encode proteins that bind to DAXX, displace ATRX, and manipulate DAXX function [27,57]. The evolution of a variety of viral mechanisms and strategies aimed at disarming ATRX/DAXX mediated restriction provides strong support for its important role as a generalized restriction factor against DNA viruses.

### ATRX delays the HSV life cycle by limiting viral DNA accessibility

The ATRX-interacting domain of DAXX is required for ATRX and DAXX-mediated restriction of HSV [19], which argues that ATRX and DAXX restrict HSV while in complex with one another. In support of this model, we showed recently that depletion of DAXX in ATRX-KO cells is not additive to ATRX knock-out in terms of HSV yield [6]. However, we also showed that ATRX is not strictly required for H3 deposition to viral DNA and that restriction of HSV by the ATRX/DAXX complex is not mediated through several of its other described functions. Taking these observations into account, we hypothesize that the ATRX/DAXX complex may inhibit DNA viruses by altering H3-containing chromatin structures.

HSV DNA in ATRX-KO cells exhibited elevated accessibility as early as 1 hpi when compared to HSV DNA in Control cells. This trend continued through 8 hpi. The accessibility of the HSV DNA increased in the HSV genomic short component repeat regions and parts of the US region from 4 to 8 hpi in both the ATRX-KO and Control cells. These areas overlapped with regions of elevated levels of viral gene expression and enriched GC content. Accessibility in the same regions was not elevated on the purified HSV DNA, suggesting that the observed changes in accessibility were not artifacts of mapping sequencing reads to repeat and GC rich regions of DNA. CpG islands and GC-rich DNA are often depleted of nucleosomes [58,59]; thus, HSV may have evolved IE genes in GC-rich regions of the genome to facilitate active transcription of those IE genes at early times of infection and to promote the efficient initiation of infection.

HSV DNA is thought to be organized into nucleosome-wrapped chromatin during latent infection [60], but the extent to which nucleosomes are associated with the HSV genome during lytic infection has been a more controversial topic. Despite the rapid deposition of histones on viral DNA upon nuclear entry, studies investigating the organization of viral chromatin with the use of nuclease digestion suggest that viral DNA is not organized into ordered nucleosomes. DNase digestion of DNA from cells during lytic HSV infection largely yielded smeared staining when probed for HSV DNA [9,61,62]. However, these same studies showed that increasing the concentration of DNase could yield a single band of approximately 150 bp, the size of a DNA fragment protected by nucleosomes, suggesting that a small fraction of viral DNA yielded fragments consistent with nucleosome protection [61,63]. While nucleosome-like structures may associate with some fraction of viral DNA, they do not do so in a regular repeating structure [9]. One study suggested that nucleosome-like structures associate only loosely with HSV genomes and that the susceptibility of HSV DNA to nuclease digestion changed during the course of infection [62]. The fragmentation of HSV DNA by the Tn5 transposase during ATAC-seq showed little evidence for nucleosome-mediated protection of viral DNA in ATRX-KO cells at 4 and 8 hpi despite the presence of high levels of H3 across the entire HSV genome. However, the distribution curve of sequenced fragment lengths is slightly enriched at 200 bp for HSV DNA in ATRX-positive Control cells at 4hpi. This may be indicative of protection from Tn5 activity by nucleosomes or nucleosome-like structures that are more stable in the presence of ATRX. This would also agree with previous reports of a minor population of nucleosome-bound HSV DNA during lytic infection and support a model in which HSV is only loosely bound in unstable nucleosomes or nucleosome-like structures [4,61–63]. There may also be a slight enrichment of 200bp fragments mapping to the HSV genome in ATRX-KO cells at 1 hpi. This may be explained by altered histone or chromatin structure and/or dynamics in the absence of ATRX. The enhanced viral kinetics observed during ATRX depletion may also contribute to this observation. By 8 hpi, the distribution of read lengths in Control cells more closely resembles the curve observed in ATRX-KO cells.

We hypothesize that ATRX promotes altered viral chromatin structure that results in reduced accessibility and prolonged histone retention on HSV DNA, thereby reducing accessibility for viral transcription and replication factors. Supporting this hypothesis, a recent study employing micrococcal nuclease digestion of HSV DNA during lytic infection coupled with short-read deep sequencing reported that increased accessibility of HSV DNA was associated with overall higher levels of viral gene expression [64]. Likewise, we previously reported that depletion of ATRX led to elevated transcription of viral genes representative of all kinetic classes [6] and report here that the overall HSV transcript level is higher in ATRX-KO cells as detected by RNA-seq. By limiting HSV genome accessibility, ATRX could effectively slow the kinetics of viral infection and reduce levels of viral gene expression as we previously observed [6]. ATRX may also be promoting the stability of nucleosomes or nucleosome-like structures, such as the previously described partially assembled nucleosome structures (PANS) [65–67], during very early stages of infection which would be likely destabilized by transcription of viral genes and the activity of ICP0.

## Materials and methods

### Cell culture, viruses, and infections

HFF cells were obtained from the American Type Culture Collection (Manassas, VA). TERT-HF immortalized fibroblasts were a kind gift from Robert Kalejta. All cells are regularly tested for the presence of mycoplasma contamination, and the cells used in this study were mycoplasma-free.

Both HFF and TERT-HF cells were maintained in Dulbecco’s Modified Eagle’s medium (DMEM; Corning, Corning NY) with 10% (v/v) fetal bovine serum (FBS) grown in humidified 5% CO_2_ incubators at 37°C. Cells were serially passaged by trypsin-EDTA (0.05%; Corning) treatment and transfer to fresh media. ATRX-KO and Control cells were maintained under 4 μg/ml puromycin.

HSV-1 KOS was utilized as the wild type virus. HSV-1 7134 and 7134R viruses [68] were used as ICP0- and ICP0-rescued viruses, respectively. Viral infection of cells was carried out at the indicated MOI in PBS containing 1% (w/v) glucose and 1% (v/v) bovine calf serum (BCS) [69]. Infections were carried out at 37°C in a shaking incubator with 1 h for adsorption time. After 1 h, the inoculum was replaced with DMEM supplemented with 1% BCS (DMEV). Infected cells were incubated at 37°C until times indicated. ICP0-null viral yields were determined by serially diluting viral lysates and infecting U2OS for a plaque assay. Cells were preincubated with flavopiridol (1 μM; Selleck Chemicals, Houston, TX), chaetocin (10 nM), or TSA (100 ng/mL) 1 h prior to infection and maintained until time of harvest in indicated experiments.

### Preparation of EdC-labeled HSV-1 stocks

EdC-labeled HSV-1 was prepared as described previously [6].

### Protein depletions

To deplete cells of endogenous proteins, siRNAs were transfected into 1x10^5^ cells plated in a 12-well dish using Lipofectamine RNAiMAX Reagent (Invitrogen). At 48 h post transfection, cells were re-seeded into assay-appropriate dishes and infected 24 h later.

siRNAs used in this study:

siNT: Dharmacon ON-TARGETplus Non-targeting pool

siATRX: Dharmacon ON-TARGETplus ATRX pool

siDAXX: Dharmacon ON-TARGETplus DAXX pool

siHIRA: Dharmacon ON-TARGETplus HIRA pool

siCHAF1A: Dharmacon ON-TARGETplus CHAF1A pool

siASF1A: Dharmacon ON-TARGETplus ASF1A pool

siASF1B: Dharmacon ON-TARGETplus ASF1B pool

siDEK: Dharmacon ON-TARGETplus DEK pool

siSSRP1: Dharmacon ON-TARGETplus SSRP1 pool

siSUV39H1: Dharmacon ON-TARGETplus SUV39H1 pool

siSETDB1: Dharmacon ON-TARGETplus SETDB1 pool

siG9A: Dharmacon ON-TARGETplus G9A pool

### Antibodies

The following antibodies were used in this study:

DAXX Sigma D7810

GAPDH Abcam ab8245

ICP8 [70]

ICP4 [71]

ICP0 East Coast Bio H1A207

ATRX Abcam 97508

H3 Abcam ab1791

H3K9me3 Abcam ab8898

Negative control rabbit IgG Millipore NG1893918 (ChIP)

HIRA Millipore 04–1488

SETDB1 Proteintech 11231-1-AP

SUV39H1 Cell Signaling 8729S

CHAF1A Cell Signaling 54805

SSRP1 Biolegend 609702

ASF1A Cell Signaling 2990S

ASF1B Cell Signaling 2769S

DEK Proteintech 16448-1-AP

MRE11 Novus NB100-142

### Immunoblots

Immunoblots were performed as previously described [6,72]. Briefly, cells were harvested at the times indicated in lithium dodecyl sulfate (LDS) sample buffer followed by incubation at 95°C for 10 min. Denatured protein samples were run on NuPAGE 4–12% bis-Tris gels (Invitrogen). Proteins were transferred from the gel to a nitrocellulose or PVDF membrane. Membranes were blocked for 1 h in LI-COR blocking solution or in a solution of 5% (wt/vol) nonfat milk in PBS containing 0.1% Tween 20 (PBST). Membranes were incubated with primary antibody overnight at 4°C followed by 3x washes with PBST. Washed membranes were incubated in secondary antibody at room temperature for 1 h (IRDye 800CW Goat anti-Mouse IgG and IRDye 800CW Goat anti-Rabbit IgG for LI-COR; α-mouse IgG^+^HRP and α-Rabbit IgG^+^HRP, Cell Signaling 7076s and 7074s, respectively for film). Membranes were imaged with a LI-COR Odyssey imager (Lincoln, NE) or were incubated with SuperSignal West Pico or Femto chemiluminescent substrate (Thermo Scientific) and imaged by film or with a chemiluminescence imager.

### Immunofluorescence and detection of EdC-labeled HSV-1 genomes

Staining of samples was performed as previously described [6]. All antibodies were used at 1:500 dilution. Images were acquired using a Nikon Ti-E inverted microscope system using a Plan Apo 100x/1.45 objective with a Zila sCMOS camera (Andor) and SPECTRA X light engine (Lumencor) controlled by NIS-Elements AR imaging software (Nikon). Image J (FIJI) was used to minimally adjust contrast of exported images.

### Chromatin Immunoprecipitation

ChIP experiments were carried out as described previously in detail [6]. Oligos for qPCR reactions:

*ICP27* Promoter (genomic)

FWD ACCCAGCCAGCGTATCCACC

REV ACACCATAAGTACGTGGC

*ICP8* promoter (genomic)

FWD GAGACCGGGGTTGGGGAATGAATC

REV CCCCGGGGGTTGTCTGTGAAGG

*GAPDH* promoter (genomic)

FWD CAGGCGCCCAATACGACCAAAATC

REV TTCGACAGTCAGTCAGCCGCATCTTCTT

### RNA-seq

For RNA-seq analysis, 2x10^6^ cells were plated into 10cm dishes. Cells were infected at an MOI of 5 and harvested at 8 hpi. Total RNA was isolated using the RNAqueous Total RNA Isolation Kit (Thermo) per the manufacturer’s instructions. The NEBNext Poly(A) mRNA Magnetic Isolation Module (NEB) was used to enrich mRNA from 1 μg of the isolated total RNA samples. Poly(A) enriched RNA was used as input for generating RNA-seq libraries using NEBNext Ultra II Directional RNA Library Prep Kit for Illumina (NEB) per the manufacturer’s instructions. Libraries were sequenced at the Bauer Core Facility at Harvard University using 2x75 paired end sequencing on an Illumina HiSeq 2500.

### ATAC-seq

For Omni-ATAC-seq analysis, 1x10^5^ TERT-HF cells were plated into 12 well plates. Cells were infected at an MOI of 5 and harvested at the times indicated. Omni-ATAC-seq was performed as described [73] with the addition of a solid phase reversible immobilization (SPRI) size selection on the final library to isolate fragments between 200–1000 bp. Libraries were sequenced at the Bauer Core Facility at Harvard University using 2x75 paired end sequencing on an Illumina HiSeq 2500.

### ChIP-seq

For ChIP-seq, 1.4x10^7^ TERT-HF cells were plated into 2x150mm dishes and were infected at an MOI of 3. Chromatin immunoprecipitation was performed as described above with the following modifications: after sonication, 300 uL of sonicated sample was added to 900 uL of IP dilution buffer. An aliquot (50 uL) of the sonicated sample was retained for input. An aliquot (6 μL) of pan-histone H3 antibody (Abcam ab1791) was added and incubated at 4°C overnight. Samples were washed as described, then eluted for 60 minutes at 65°C. The eluate was transferred to a new tube, and the eluate was incubated at 65°C to reverse crosslinking. Samples were then treated with RNase A (Ambion) for 1 h at 37°C and Proteinase K treatment (Roche) at 45°C for 2 h. DNA was purified using a QIAquick PCR purification kit (Qiagen) and eluted with 50 μl of buffer EB. An aliquot (10ng) of recovered ChIP DNA was used to prepare a library with the TruSeq ChIP Library Preparation Kit (Illumina) per the manufacturer’s instructions. Libraries were sequenced at the Bauer Core Facility at Harvard University using 2x75 paired end sequencing on an Illumina HiSeq 2500.

### Data analysis

A human-HSV genome was generated by concatenating the HSV-1 genome (HSV-1 KOS strain, GenBank accession number KT899744) [74] to the end of the human hg38 genome. Read alignments were performed as detailed below, and genome tracks were generated with Gviz [75]. Percent HSV reads of total reads was calculated using SAMtools [76].

RNA-seq: Reads were aligned to the human-HSV genome and counted using STAR [77] with ENCODE standard options for the long RNA-seq pipeline. Batch effect correction was performed using ComBat [78] and differential expression was calculated with DESeq2 [79]. Volcano plots were generated with ggplot2 [80].

ChIP-seq: Reads were aligned and peaks were called using the ENCODE pipeline for histone ChIP-seq (https://github.com/ENCODE-DCC/chip-seq-pipeline2). Samples were normalized by total reads prior to peak calling. Differential peak analysis was performed using csaw [81]. To generate ChIP-seq data tracks across HSV genome, samples were aligned with multimapping set to (bowtie2 k = 4) and normalized by total HSV reads before visualization.

ATAC-seq: Reads were aligned and peaks were called using the ENCODE pipeline for ATAC-seq with multimapping set to 0 (bowtie2 k = 1) (https://github.com/ENCODE-DCC/atac-seq-pipeline). Samples were normalized by library complexity and differential accessibility was calculated using csaw as previously described [82]. To generate ATAC-seq data tracks across the HSV genome, samples were normalized by total HSV reads before visualization. Fragment length distribution was visualized using the Galaxy platform (https://usegalaxy.org/).

GC content: The GC content across the HSV genome was calculated in 1000 bp windows offset by 100 bp using the EMBOSS isochore online tool [83]. To compare GC content with ATAC-seq counts, total HSV read-normalized ATAC-seq reads were binned into 1000 bp windows and total counts within each window was determined using edgeR [84]. The Kendall rank coefficient (tau) was calculated to determine the correlation between GC content and ATAC-seq counts.

## Supporting information

S1 Fig ATRX degradation and aggregation in the presence of HSV protein ICP0. (A) HSV protein ICP0 promotes ATRX aggregate formation post PML-NB dispersion. HFFs were infected with HSV 7134 or 7134R (7134 rescued for ICP0 expression) at MOI 5. Total protein was harvested at times indicated followed by immunoblot detection of ATRX, ICP4, ICP0, and GAPDH. (B) HFFs were infected with KOS-EdC at MOI 5. Cells were fixed by 2% formaldehyde at times indicated followed by antibody staining for ATRX and PML. Click chemistry was used to biotinylate HSV DNA followed by detection by a fluorophore-conjugated streptavidin probe. Cells were imaged at 100x. (C) Quantification of ATRX-aggregates in imaged cells. A total of 184 infected cells from 2 independent experiments were used to assess the prevalence of ATRX aggregates in fibroblasts infected with KOS-EdC at MOI 5. D) HFFs infected with HSV 7134 were fixed at 6 hpi and stained with antibodies for ATRX and ICP8. Cells were imaged at 100x.
(TIF)

S2 Fig ChIP-qPCR detection of H3 at HSV *ICP27* and *ICP8* gene promoters during infection in cells depleted for H3 chaperones. TERT-HF cells were infected with HSV 7134 at an MOI of 3. Infected cells were fixed and harvested 8 hpi. ChIP-qCPR using a pan-H3 antibody and HSV specific primers were used to detect enrichment of H3 at viral gene promoters for (A) *ICP27* and (B) *ICP8*. Results reported as Relative IP (the percent of input immunoprecipitated by each antibody normalized to the 8-hour control sample—set to 1.0 for each replicate). (C) Chromatin input for ICP8 relative to input GAPDH to determine relative viral genome copy numbers. Results were analyzed by one sample T-test. Data are reported as the average of 3 independent experiments, with the exception of HIRA single depletion results which have 2 replicates due to mechanical failure during sample processing. (D) TERT-HFs were treated with siRNAs against non-targeting (NT), ATRX, HIRA, and DEK and infected with HSV 7134 at an MOI of 5 in the (D) absence or (E) presence of acyclovir (ACV). Relative viral transcripts for *ICP27*, *ICP8*, or *gB* were quantified by qPCR at 8 hpi. Viral mRNA levels were normalized to cellular 18S transcripts. Results were analyzed by One-way ANOVA using Dunnet’s multiple comparison correction. Data are reported as the average of 3 independent experiments ± standard error of the mean; p < 0.05 (*), p < 0.01 (**), p < 0.001 (***).
(TIF)

S3 Fig ICP0-null viral yields in cells depleted of histone methyltransferases SUV39H1 and SETDB1. (A) Immunoblot of lysates from TERT-HF cells treated with siRNA against non-targeting (NT), ATRX, SUV39H1, and SETDB1. (B) Viral yields from TERT-HF cells treated with siRNAs against non-targeting, ATRX, SUV39H1, and SETDB1 which were infected with HSV 7134 at an MOI of 0.1. Viral lysates were collected at 48 hpi and titrated on U2OS cells. Yields were normalized to (PFU/mL)/1x10^5^ cells. Results were analyzed by One-way ANOVA using Dunnet’s multiple comparison correction. (C) Immunoblot detection of ATRX in Control and ATRX-KO cell lines. ATRX-KO #2 was used in this study. (D) Viral yields from ATRX-KO and Control cells which were infected with HSV 7134 at an MOI of 0.1 in the presence of SUV39H1 inhibitor, chaetocin, following a 1-hour pre-treatment. Viral lysates were collected at 48 hpi and titrated on U2OS cells. Yields were normalized to (PFU/mL)/1x10^5^ cells. Viral yield was determined by infecting U2OS cells with serial dilutions of harvested viral lysates. Data are reported as the average of 3 independent experiments ± standard error of the mean; p < 0.05 (*), p < 0.01 (**), p < 0.001 (***).
(TIF)

S4 Fig ICP0- yields during infection of cells treated with HDAC inhibitor. (A) Viral yields from ATRX-KO and Control cells which were infected with HSV 7134 at an MOI of 0.1 in the presence of an HDAC class I and II inhibitor, trichostatin A (TSA) following a 1-hour pre-treatment. Viral lysates were collected at 48 hpi and titrated on U2OS cells. Yields were normalized to (PFU/mL)/1x10^5^ cells. Data are reported as the average of 3 independent experiments ± standard error of the mean; p < 0.05 (*), p < 0.01 (**), p < 0.001 (***).
(TIF)

S5 Fig HSV gene expression during infection of ATRX-KO and Control cells. RNA-seq read coverage of the HSV genome from poly(A) enriched RNA harvested at 8 hpi from ATRX-KO and Control cells infected with either KOS or 7134 HSV strains at an MOI of 5. Samples were normalized by total human + HSV reads to account for differences in sequencing depth prior to visualization.
(TIF)

S6 Fig Differential gene expression during HSV ICP0-null infection of ATRX-KO and Control cells. Venn diagrams of genes (A) downregulated and (B) upregulated in ATRX-KO and Control cells infected with HSV 7134 at 8 hpi. Expression of MAMDC2-AS1 antisense transcript in uninfected and 7134 infected ATRX-KO and Control cells. Normalized RNA-seq read counts of MAMDC2-AS1 antisense transcript in (C) uninfected and (D) 7134 infected (MOI 5, 8 hpi) ATRX-KO and Control cells.
(TIF)

S7 Fig ChIP-seq at the H2AX human gene locus and the UL region of HSV during infection. ChIP-seq reads for H3 were visualized with the Integrative Genome Viewer (https://igv.org/) for the H2AX gene locus of the human genome and part of the UL region of the HSV genome 4 hpi with HSV 7134 at MOI 3.
(TIF)

S8 Fig Correlation analysis of ATAC-seq signal to GC content in the HSV US and flanking repeat regions. GC content and normalized ATAC-seq counts for proteinase K-treated KOS genomic DNA, ATRX-KO and Control cells at 8 hpi with HSV 7134 were calculated in 1000 bp windows and the HSV US and flanking repeat regions were plotted. The ATAC-seq vs GC content for reads that map to the HSV US and flanking repeat regions in (A) ATRX-KO cells 8 hpi, (B) Control cells 8 hpi, and (C) purified proteinase K treated HSV KOS DNA. Plots show the ATAC-seq read vs GC content for 2 replicates. (D) The Kendall rank coefficient (tau) was calculated to determine the correlation between GC content and ATAC-seq counts in ATRX-KO 8hpi, Control 8 hpi, and KOS DNA.
(TIF)

S9 Fig Frequency distribution of ATAC-seq DNA read lengths during HSV infection, additional replicates. Sequenced fragment length distribution for ATAC-seq reads mapped to the HSV genomes in Control and ATRX-KO cells (A) 1 and (B) 4 hpi with 7134.
(TIF)

S1 Table Differential expression analysis of sense transcripts. Reads were mapped to a human (hg38) + HSV genome and read counts for each gene was calculated using STAR. ComBat was used to correct for batch effect and DESeq2 was used to normalize counts and calculate differential expression for reads that map to the sense strand. Genes with an adjusted p value < 0.05 and a fold change > 2 or < -2 are reported. ATRX_UP: Genes upregulated in ATRX KO cells infected with HSV 7134 at 8 hpi compared to uninfected ATRX KO cells. ATRX_DOWN: Genes downregulated in ATRX KO infected with HSV 7134 at 8 hpi cells compared to uninfected ATRX KO cells. CTRL_UP: Genes upregulated in Control cells infected with HSV 7134 at 8 hpi compared to uninfected Control cells. CTRL_DOWN: Genes downregulated in Control cells infected with HSV 7134 at 8 hpi compared to uninfected Control cells. inf_UP: Genes upregulated in ATRX KO cells infected with HSV 7134 at 8 hpi compared to Control cells infected with HSV 7134 at 8 hpi. inf_DOWN: Genes downregulated in ATRX KO cells infected with HSV 7134 at 8 hpi compared to Control cells infected with HSV 7134 at 8 hpi. No genes met the p value and fold change cutoff, so this list is not reported. uninf_UP: Genes upregulated in uninfected ATRX KO cells compared to uninfected Control cells. uninf_DOWN: Genes downregulated in uninfected ATRX KO cells compared to uninfected Control cells.
(XLSX)

S2 Table Differential expression analysis of antisense transcripts. Reads were mapped to a human (hg38) + HSV genome and read counts for each gene was calculated using STAR. ComBat was used to correct for batch effect and DESeq2 was used to normalize counts and calculate differential expression for reads that map to the antisense strand. Genes with an adjusted p value < 0.05 and a fold change > 2 are reported. See S1 Table legend for explanation of gene list labels. No genes met the p value and fold change cutoffs for inf_DOWN, uninf_UP, or uninf_DOWN so these lists are not reported.
(XLSX)
