# Supplementary figures and images for: ATRX limits the accessibility of histone H3-occupied HSV genomes during lytic infection
Source: PLoS Pathog. 2021 Apr 28;17(4):e1009567. doi: 10.1371/journal.ppat.1009567 (PMC8109836; doi:10.1371/journal.ppat.1009567)

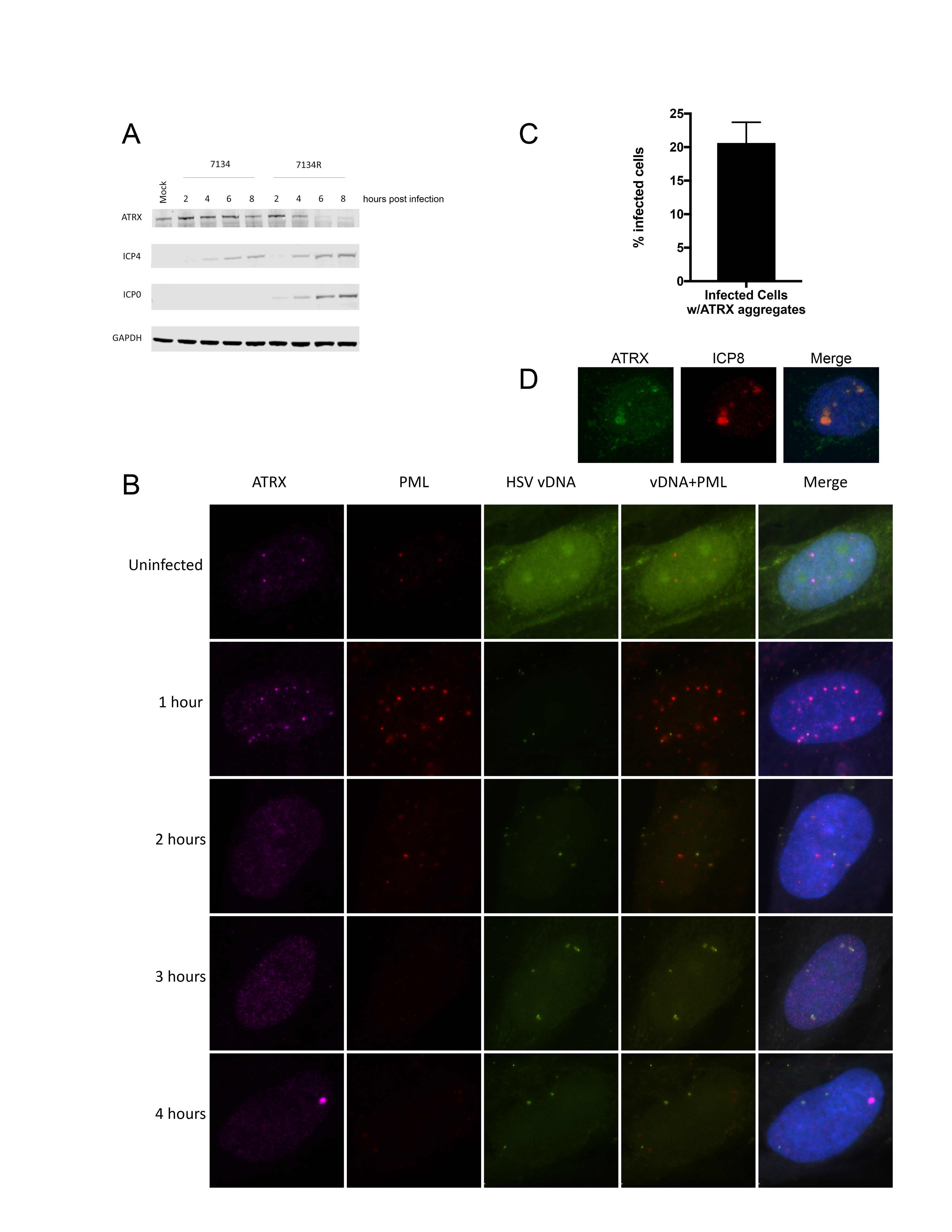

Supplement: S1 Fig — (A) HSV protein ICP0 promotes ATRX aggregate formation post PML-NB dispersion. HFFs were infected with HSV 7134 or 7134R (7134 rescued for ICP0 expression) at MOI 5. Total protein was harvested at times indicated followed by immunoblot detection of ATRX, ICP4, ICP0, and GAPDH. (B) HFFs were infected with KOS-EdC at MOI 5. Cells were fixed by 2% formaldehyde at times indicated followed by antibody staining for ATRX and PML. Click chemistry was used to biotinylate HSV DNA followed by detection by a fluorophore-conjugated streptavidin probe. Cells were imaged at 100x. (C) Quantification of ATRX-aggregates in imaged cells. A total of 184 infected cells from 2 independent experiments were used to assess the prevalence of ATRX aggregates in fibroblasts infected with KOS-EdC at MOI 5. D) HFFs infected with HSV 7134 were fixed at 6 hpi and stained with antibodies for ATRX and ICP8. Cells were imaged at 100x. (TIF) [file ppat.1009567.s001.tif]

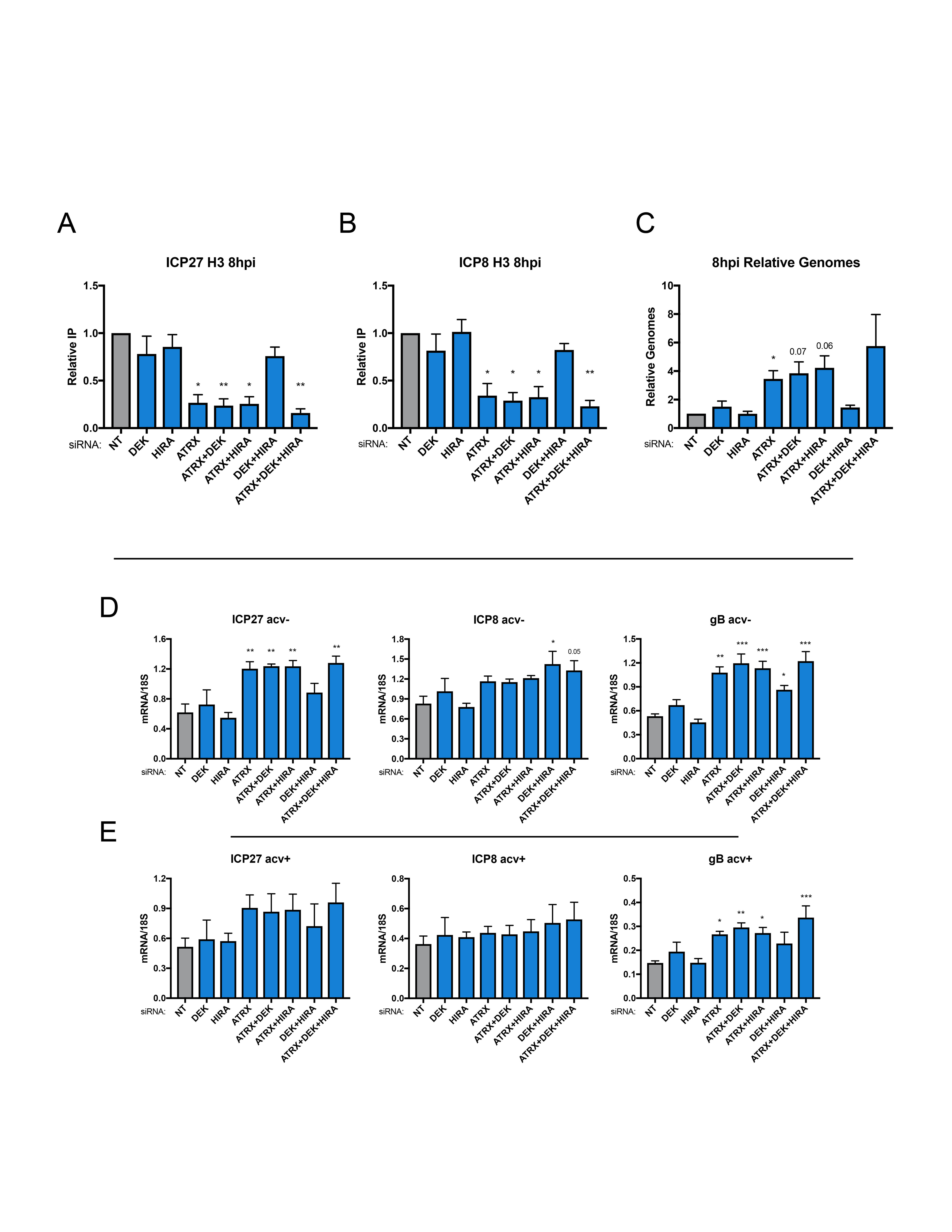

Supplement: S2 Fig — TERT-HF cells were infected with HSV 7134 at an MOI of 3. Infected cells were fixed and harvested 8 hpi. ChIP-qCPR using a pan-H3 antibody and HSV specific primers were used to detect enrichment of H3 at viral gene promoters for (A) ICP27 and (B) ICP8. Results reported as Relative IP (the percent of input immunoprecipitated by each antibody normalized to the 8-hour control sample—set to 1.0 for each replicate). (C) Chromatin input for ICP8 relative to input GAPDH to determine relative viral genome copy numbers. Results were analyzed by one sample T-test. Data are reported as the average of 3 independent experiments, with the exception of HIRA single depletion results which have 2 replicates due to mechanical failure during sample processing. (D) TERT-HFs were treated with siRNAs against non-targeting (NT), ATRX, HIRA, and DEK and infected with HSV 7134 at an MOI of 5 in the (D) absence or (E) presence of acyclovir (ACV). Relative viral transcripts for ICP27, ICP8, or gB were quantified by qPCR at 8 hpi. Viral mRNA levels were normalized to cellular 18S transcripts. Results were analyzed by One-way ANOVA using Dunnet’s multiple comparison correction. Data are reported as the average of 3 independent experiments ± standard error of the mean; p < 0.05 (*), p < 0.01 (**), p < 0.001 (***). (TIF) [file ppat.1009567.s002.tif]

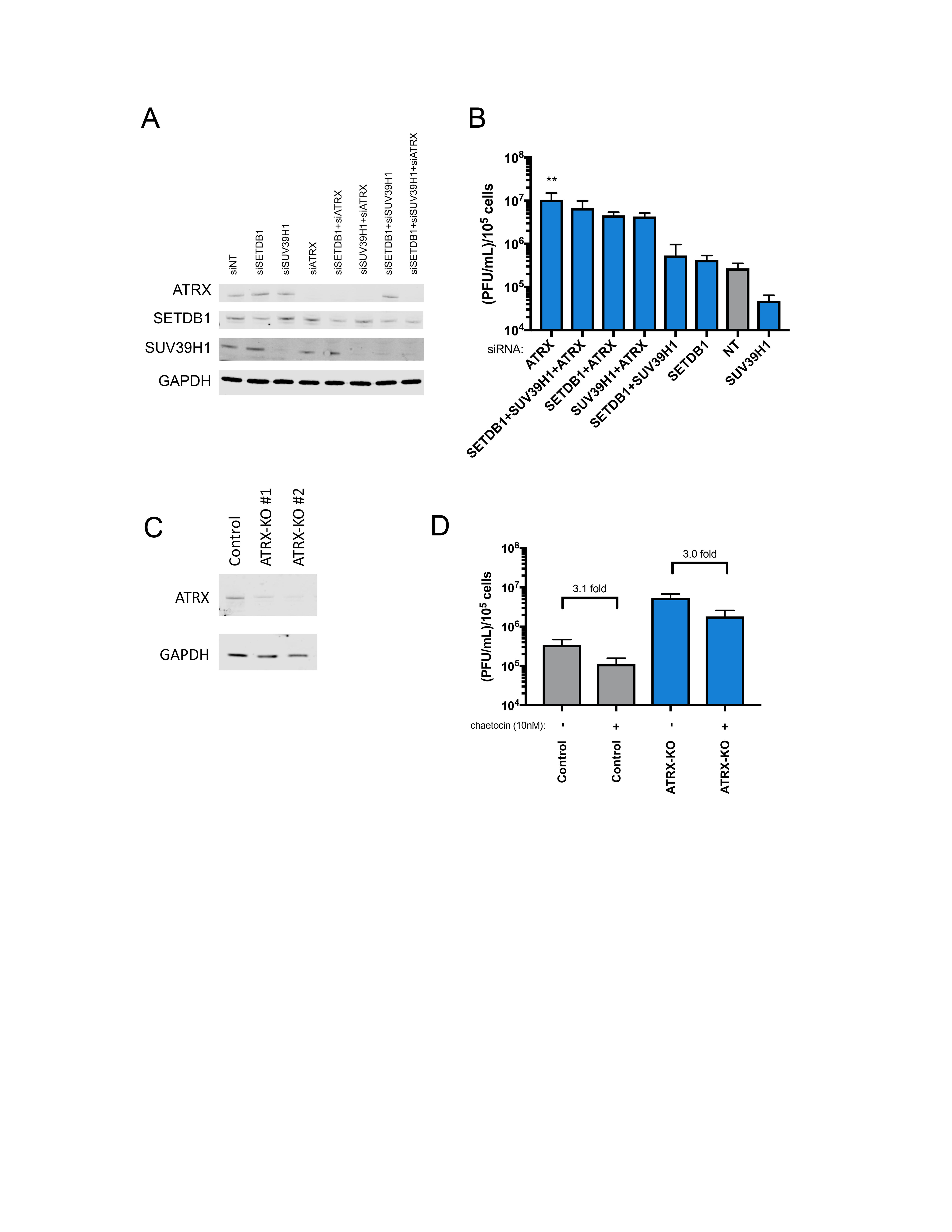

Supplement: S3 Fig — (A) Immunoblot of lysates from TERT-HF cells treated with siRNA against non-targeting (NT), ATRX, SUV39H1, and SETDB1. (B) Viral yields from TERT-HF cells treated with siRNAs against non-targeting, ATRX, SUV39H1, and SETDB1 which were infected with HSV 7134 at an MOI of 0.1. Viral lysates were collected at 48 hpi and titrated on U2OS cells. Yields were normalized to (PFU/mL)/1x105 cells. Results were analyzed by One-way ANOVA using Dunnet’s multiple comparison correction. (C) Immunoblot detection of ATRX in Control and ATRX-KO cell lines. ATRX-KO #2 was used in this study. (D) Viral yields from ATRX-KO and Control cells which were infected with HSV 7134 at an MOI of 0.1 in the presence of SUV39H1 inhibitor, chaetocin, following a 1-hour pre-treatment. Viral lysates were collected at 48 hpi and titrated on U2OS cells. Yields were normalized to (PFU/mL)/1x105 cells. Viral yield was determined by infecting U2OS cells with serial dilutions of harvested viral lysates. Data are reported as the average of 3 independent experiments ± standard error of the mean; p < 0.05 (*), p < 0.01 (**), p < 0.001 (***). (TIF) [file ppat.1009567.s003.tif]

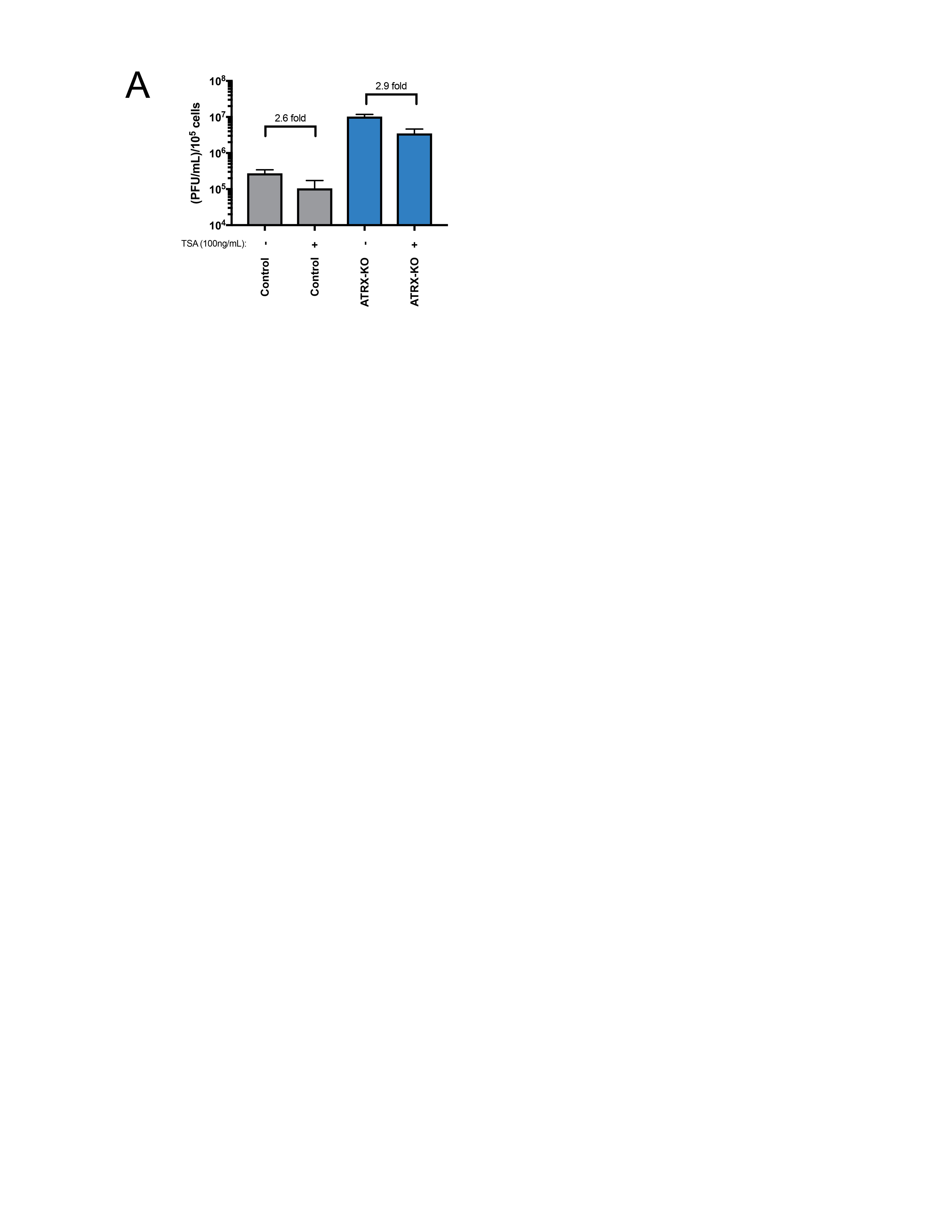

Supplement: S4 Fig — (A) Viral yields from ATRX-KO and Control cells which were infected with HSV 7134 at an MOI of 0.1 in the presence of an HDAC class I and II inhibitor, trichostatin A (TSA) following a 1-hour pre-treatment. Viral lysates were collected at 48 hpi and titrated on U2OS cells. Yields were normalized to (PFU/mL)/1x105 cells. Data are reported as the average of 3 independent experiments ± standard error of the mean; p < 0.05 (*), p < 0.01 (**), p < 0.001 (***). (TIF) [file ppat.1009567.s004.tif]

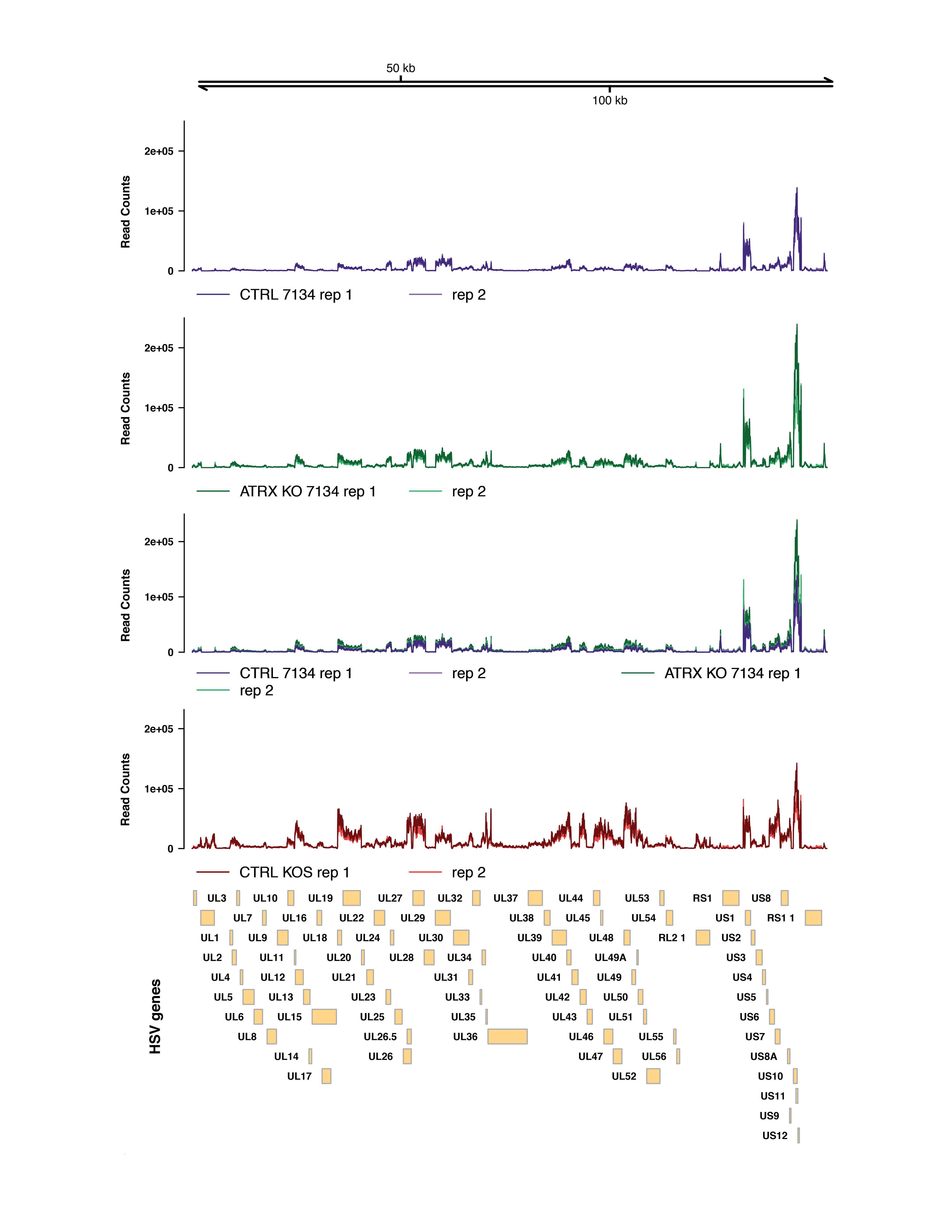

Supplement: S5 Fig — RNA-seq read coverage of the HSV genome from poly(A) enriched RNA harvested at 8 hpi from ATRX-KO and Control cells infected with either KOS or 7134 HSV strains at an MOI of 5. Samples were normalized by total human + HSV reads to account for differences in sequencing depth prior to visualization. (TIF) [file ppat.1009567.s005.tif]

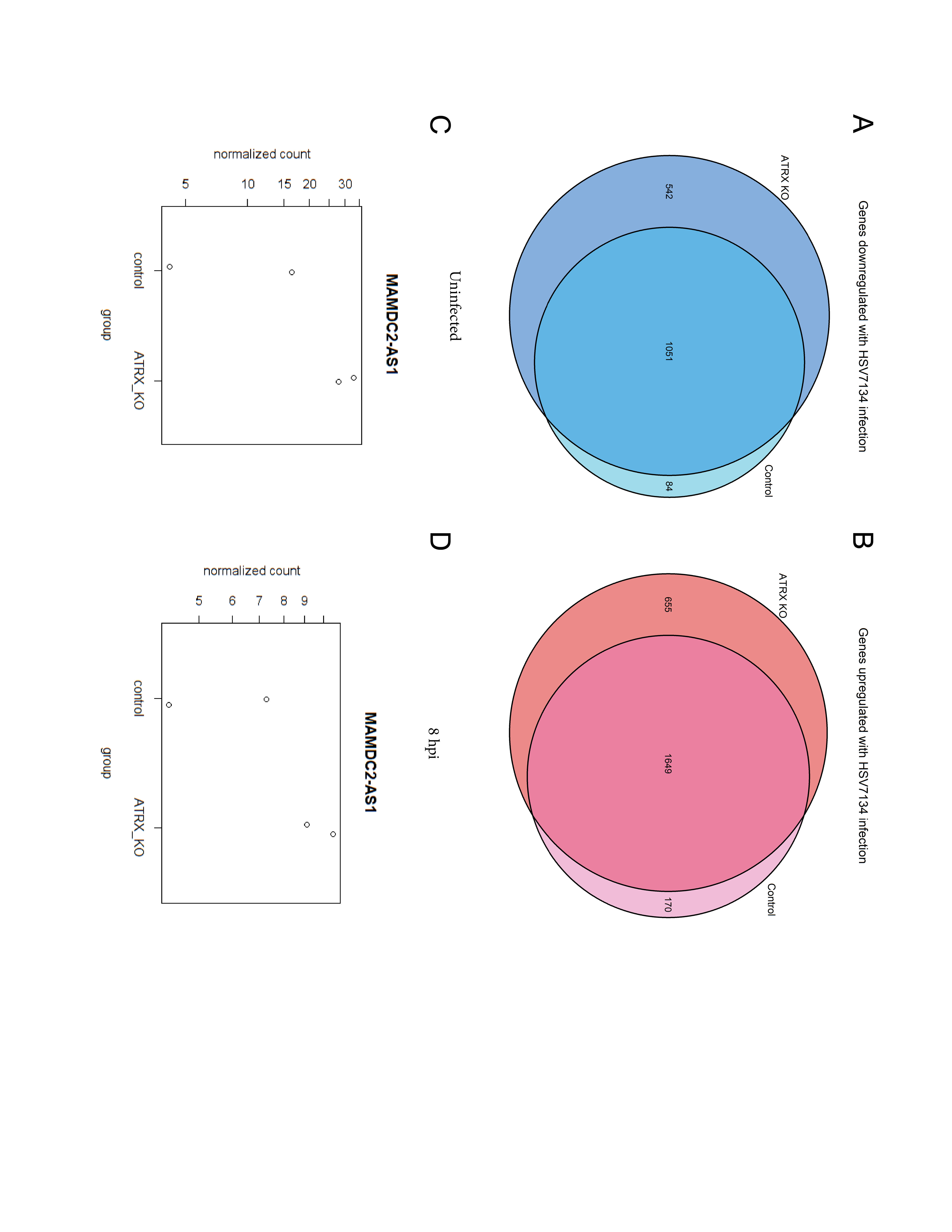

Supplement: S6 Fig — Venn diagrams of genes (A) downregulated and (B) upregulated in ATRX-KO and Control cells infected with HSV 7134 at 8 hpi. Expression of MAMDC2-AS1 antisense transcript in uninfected and 7134 infected ATRX-KO and Control cells. Normalized RNA-seq read counts of MAMDC2-AS1 antisense transcript in (C) uninfected and (D) 7134 infected (MOI 5, 8 hpi) ATRX-KO and Control cells. (TIF) [file ppat.1009567.s006.tif]

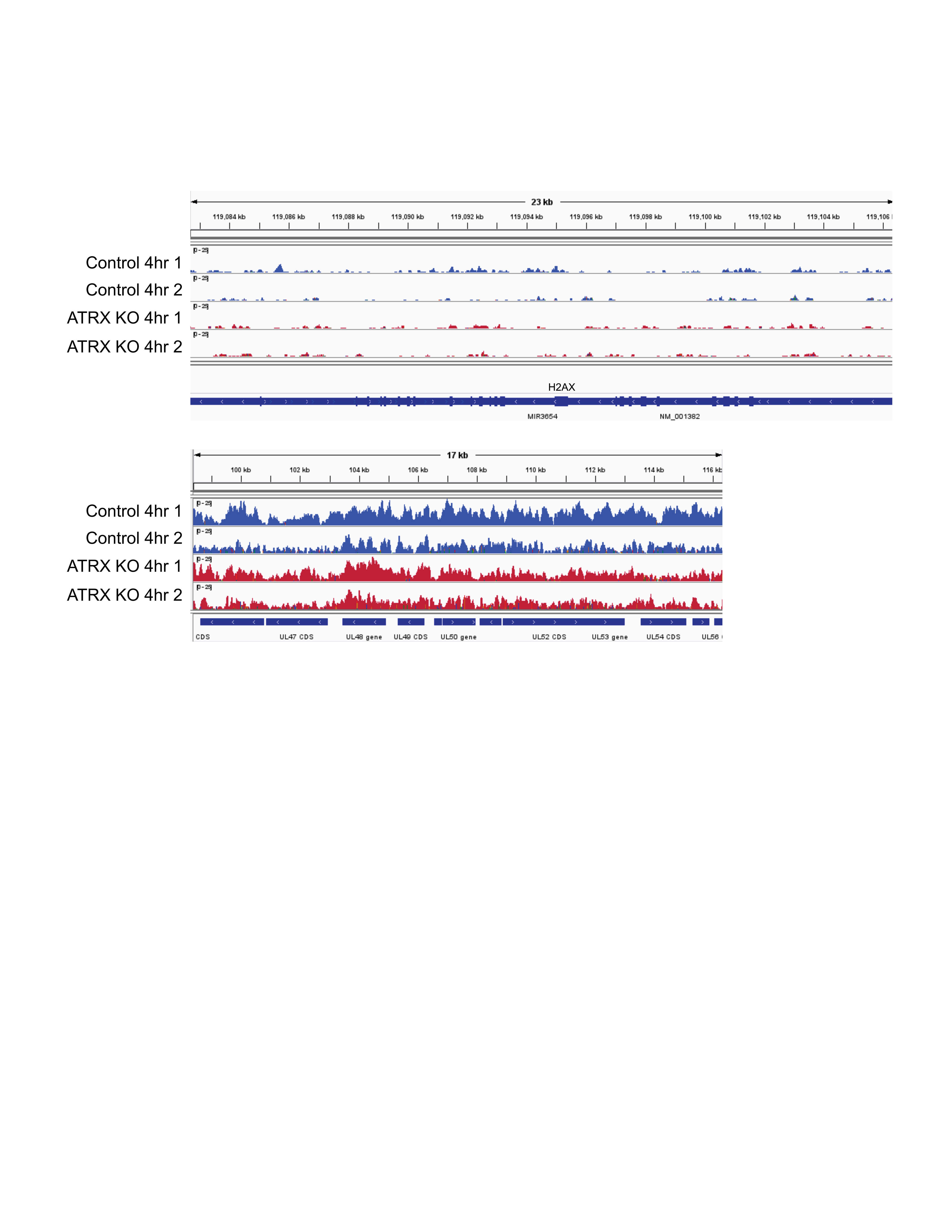

Supplement: S7 Fig — ChIP-seq reads for H3 were visualized with the Integrative Genome Viewer (https://igv.org/) for the H2AX gene locus of the human genome and part of the UL region of the HSV genome 4 hpi with HSV 7134 at MOI 3. (TIF) [file ppat.1009567.s007.tif]

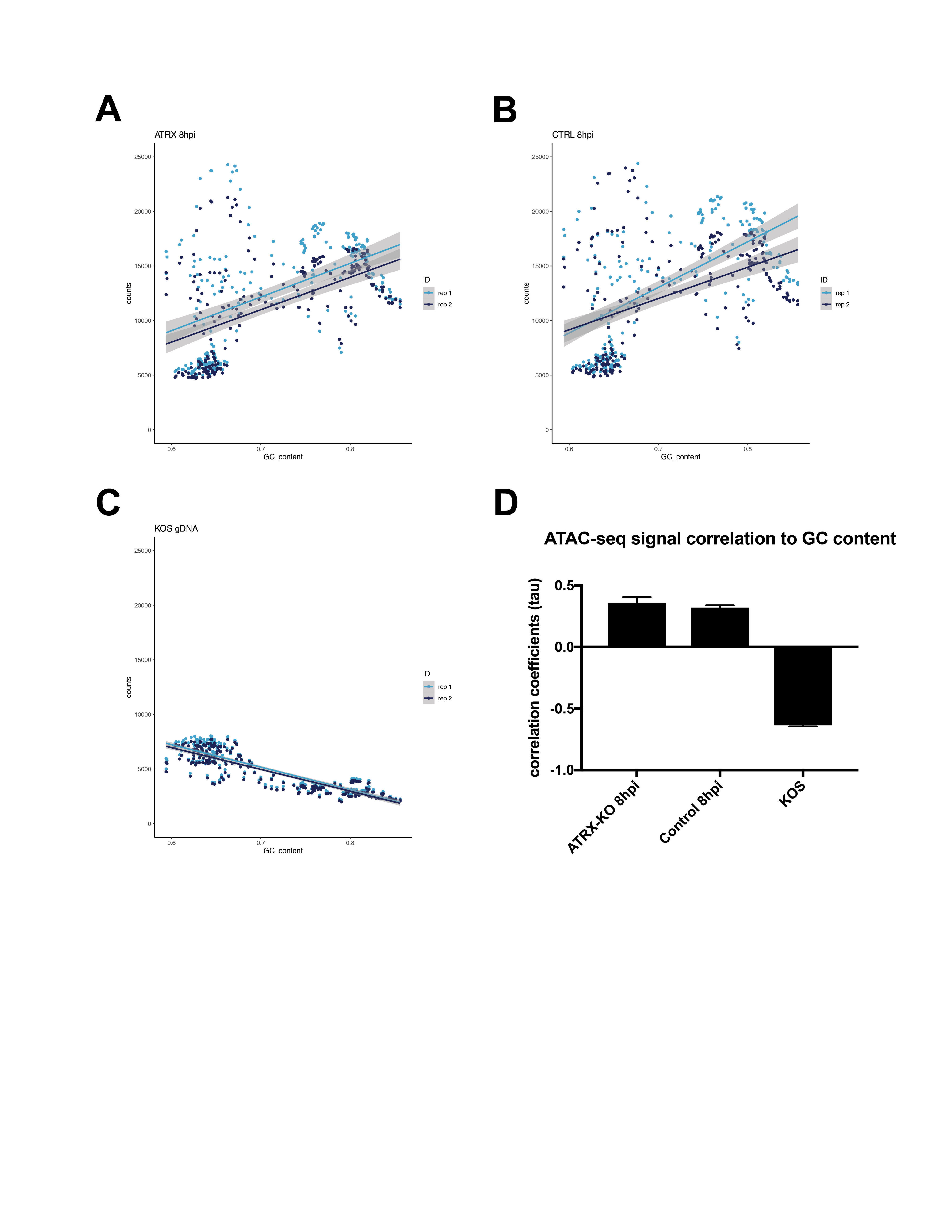

Supplement: S8 Fig — GC content and normalized ATAC-seq counts for proteinase K-treated KOS genomic DNA, ATRX-KO and Control cells at 8 hpi with HSV 7134 were calculated in 1000 bp windows and the HSV US and flanking repeat regions were plotted. The ATAC-seq vs GC content for reads that map to the HSV US and flanking repeat regions in (A) ATRX-KO cells 8 hpi, (B) Control cells 8 hpi, and (C) purified proteinase K treated HSV KOS DNA. Plots show the ATAC-seq read vs GC content for 2 replicates. (D) The Kendall rank coefficient (tau) was calculated to determine the correlation between GC content and ATAC-seq counts in ATRX-KO 8hpi, Control 8 hpi, and KOS DNA. (TIF) [file ppat.1009567.s008.tif]

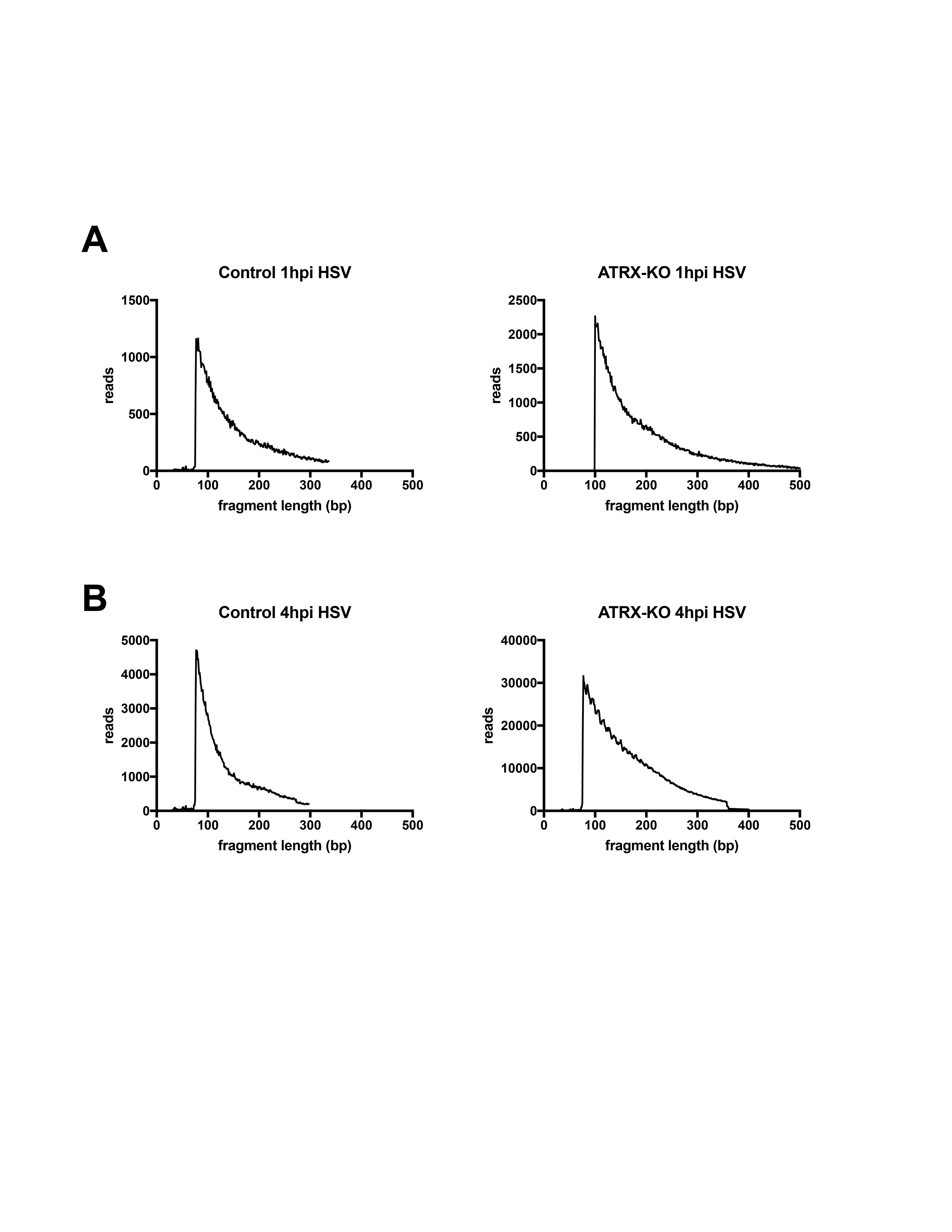

Supplement: S9 Fig — Sequenced fragment length distribution for ATAC-seq reads mapped to the HSV genomes in Control and ATRX-KO cells (A) 1 and (B) 4 hpi with 7134. (TIF) [file ppat.1009567.s009.tif]
